# Virulence and Stress-Related Proteins Are Differentially Enriched and *N*-Terminally Acetylated in Extracellular Vesicles from Virulent *Paracoccidioides brasiliensis*

**DOI:** 10.3390/jof11100751

**Published:** 2025-10-21

**Authors:** Carla E. Octaviano-Azevedo, Karolina R. F. Beraldo, Natanael P. Leitão-Júnior, Cássia M. de Souza, Camila P. da Silva, Rita C. Sinigaglia, Erix A. Milán Garcés, Evandro L. Duarte, Alexandre K. Tashima, Maria A. Juliano, Rosana Puccia

**Affiliations:** 1 Departamento de Microbiologia, Imunologia e Parasitologia, Escola Paulista de Medicina, Universidade Federal de São Paulo, São Paulo 04023-062, SP, Brazil; 2 Departamento de Biofísica, Escola Paulista de Medicina, Universidade Federal de São Paulo, São Paulo 04044-020, SP, Brazil; karolina.rosa@unifesp.br (K.R.F.B.);; 3 Instituto Carlos Chagas, Fundação Oswaldo Cruz, Curitiba 81350-010, PR, Brazil; 4 Centro de Microscopia Eletrônica, Escola Paulista de Medicina, Universidade Federal de São Paulo, São Paulo 04023-062, SP, Brazil; 5 Departamento de Física Geral, Instituto de Física, Universidade de São Paulo, São Paulo 05508-090, SP, Brazil; garces@usp.br (E.A.M.G.); elduarte@usp.br (E.L.D.); 6 Departamento de Bioquímica, Escola Paulista de Medicina, Universidade Federal de São Paulo, São Paulo 04023-901, SP, Brazil; aktashima@unifesp.br

**Keywords:** extracellular vesicles, proteome, *Paracoccidioides brasiliensis*, virulent/attenuated/stressed Pb18, virulence factors

## Abstract

Extracellular vesicles (EVs) are bilayer-membrane cellular components that deliver protected cargo to the extracellular environment and can mediate long-distance signaling. We have previously reported that EVs isolated from the virulent fungal pathogen *Paracoccidioides brasiliensis* Vpb18 can revert the expression, in the attenuated variant Apb18, of stress-related virulence traits. We presently show that the Vev and Aev, respectively, produced by these variants display distinct proteomes, with prevalent functional enrichment in Vev related to oxidative stress response, signal transduction, transport, and localization, in addition to richer protein–protein interaction. Proteome sequences were obtained by nanoflow liquid chromatography coupled with tandem mass spectrometry (nano LC-ESI-MS/MS). The Vev and corresponding Vpb18 proteomes also differed, suggesting a selective bias in vesicle protein cargo. Moreover, sublethal oxidative (VevOxi) and nitrosative (VevNO) stress modulated the Vev proteome and a positive correlation between VevOxi/VevNO-enriched and Vev-enriched (relative to Aev) proteins was observed. Out of 145 fungal virulence factors detected in Vev, 64% were enriched, strongly suggesting that molecules with virulence roles in *Paracoccidioides* are selectively concentrated in Vev. Our study significantly advanced the field by exploring protein *N*-terminal acetylation to a dimension rarely investigated in fungal EV proteomics. The proportion of *N*-terminally acetylated proteins in Vev was higher than in Vpb18 and the presence of Nt-acetylation in Vev-enriched virulence factors varied across the samples, suggesting that it might interfere with protein sorting into EVs and/or protein functionality. Our findings highlight the relevance of our fungal model to unraveling the significance of fungal EVs in pathogenesis and phenotypic transfer.

## 1. Introduction

Extracellular vesicles (EVs) are bilayer lipid membrane components released by the cells from all kingdoms and which cannot replicate independently [1]. According to the Minimal Information for Studies of Extracellular Vesicles [1], the term extracellular vesicles encompasses small (<200 nm) and large particles derived from the endosomal compartment (exosomes) or cell surface (ectosomes or microvesicles). EVs are involved in the delivery of protected cargo to the extracellular environment and therefore they can mediate long-distance signaling between pathogen and hosts or other microorganisms [2]. The studies on their relevance to cell–cell communication, cell wall remodeling, and virulence in medically important fungal species has been growing exponentially during the last years [3,4]. The fungal EV cargo includes hundreds of proteins, peptides, small and messenger RNA species, polysaccharides, oligosaccharides, pigment, and membrane lipids [5].

Our group and collaborators have contributed to unraveling the *Paracoccidioides* spp. EV secretome, carbohydrate cargo and surface oligosaccharide ligands, lipidome, and RNA content [6]. These publications provided detailed comparisons of the EV cargo from isolates representing different *Paracoccidioides* species and from other fungal species. As an example, the *P. brasiliensis* Pb18 secretome included both EV and EV-free fractions. These data were compared with EV proteomes available at the time for *Cryptococcus neoformans*, *Histoplasma capsulatum*, *Candida albicans*, and *Saccharomyces cerevisiae*, showing 63% overlap among EV orthologs [7]. Twenty-six proteins were common to all species, including enzymes involved in the fungal defense against oxidative stress and other potential virulence regulators. In addition, 60% of the *Paracoccidioides* cell wall proteomes overlapped with fungal EV proteins [8].

*Paracoccidioides* species are thermo-dependent dimorphic fungi that cause paracoccidioidomycosis (PCM) in the yeast phase [9,10]. PCM affects the lungs especially, where the disease progression depends on the first encounter with the cells of the innate immune system and subsequent adaptative response, which ultimately protects against the disease [11]. Reactive oxygen (ROS) and nitrogen (NOS) species of the respiratory burst produced by the immune cells are key effector mechanisms in fungal clearance by the granuloma [11,12,13]

The *P. brasiliensis* Pb18 isolate has been broadly studied because of its virulence in mice [14]. Pb18 EVs stimulate the expression of proinflammatory mediators by murine peritoneal macrophages and are able to revert the macrophage phenotype from M2 to M1, stimulating high fungicidal activity [15,16]. Baltazar et al. [17] managed to protect mice against PCM by inoculating Pb18 EVs before fungal challenge, while our group reported the enhancement of the infection prompted by EVs [16]. Montanari Borges et al. [18] showed that Pb18 EVs produced by yeasts recently isolated from mice organs evoked milder adaptative immune response than EVs from the slightly less virulent yeasts used in mouse infection.

Considering the constraints posed by the *Paracoccidioides* genetic manipulation [19], the classical virulent/attenuated Pb18 model of *P. brasiliensis*, which is distinct from that used by Montanari Borges et al. [18], proved to be useful to evaluate virulence regulators [20,21]. The virulent/attenuated model consists of Pb18 variants transiently displaying either high virulence or highly attenuated virulence capacity in mice. In our laboratory, virulent Pb18 yeasts have uninterruptedly been recovered from mice organs and the highly attenuated variant has been subcultured in vitro for years [16]. Enzymes associated with protection to oxidative stress are more abundant in the virulent than in the attenuated variant, as revealed by comparative cell proteome, but virulence and resistance to oxidative stress can be recovered after two passages of the attenuated variant in mice [21].

We have previously managed to recover resistance to oxidative stress of the highly attenuated Pb18 variant (here called Apb18) after incubation with Vev extracellular vesicles isolated from virulent Vpb18 [16]. Higher resistance to stress was probably due to the observed increase in gene expression of antioxidant molecules like alternative oxidase AOX, peroxiredoxins HYR1 and PRX1, in addition to higher catalase activity. We showed that Aev vesicles, isolated from attenuated Apb18, stimulated the expression of higher levels of inflammatory mediators both in vivo and in vitro, which probably contributed to the exacerbated *P. brasiliensis* murine infection especially associated with Aev-treated animals [16].

In order to understand how extracellular vesicle proteins from the *P. brasiliensis* Pb18 virulent/attenuated variants could differentially contribute to pathogenesis, we compared the Vev and Aev vesicle proteomes, the parent yeast cell extract proteins, and the protein cargo from Vev derived from Vpb18 cultivated under sublethal oxidative and nitrosative stress. Importantly, we addressed differences in post-translational modifications rarely observed in the fungal extracellular vesicle literature.

## 2. Materials and Methods

### 2.1. Fungal Strains and Culture Conditions

The fungal working model was the yeast phase from the *P. brasiliensis* virulent isolate Vpb18 and its attenuated variant Apb18 [16,20]. The cultures were stored in mYPD slants (0.5% yeast extract, 1% casein peptone, 0.5% glucose, pH 6.5) at 4 °C for up to three months. For experimental procedures, the stored cultures were recovered in fresh mYPD solid medium at 36 °C for 6 to 7 days, and then seeded in 200 mL of defined Ham’s F-12 medium (Life Technologies, Grand Island, NY, USA) supplemented with 0.5% glucose (Ham/glc), under agitation (120 rpm) for 4 days (log-phase pre-inoculum). For EV preparation, confluent yeast cultures in Ham/glc agar plates were prepared using the cells from this pre-inoculum, as detailed previously [16].

### 2.2. Vev and Aev Preparation

Vev and Aev were prepared from confluent cultures [22] grown in Petri dishes (90 × 15 mm) containing Ham/glc agar, as detailed in our previous publication [16]. Briefly, yeast cells from the pre-inoculum mentioned previously were precipitated by centrifugation, spread in Ham/glc agar (9 × 10^6^ viable cells/600 µL/plate) and incubated for 2 days at 36 °C. The growth from three plates, corresponding to one EV preparation, was gently scrapped and transferred to a conic tube containing 30 mL of sterile PBS (phosphate-buffered saline). The suspension was vortexed gently to liberate the EVs to the buffer and centrifuged at 4000× *g* for 15 min at 4 °C. The supernatant was centrifuged for 30 min at 15,000× *g*, filtered through a 0.45-μm membrane and ultracentrifuged at 100,000× *g* for 1 h, at 4 °C [7,16,22]. The EV-containing pellet was washed in PBS and suspended in 300 mL PBS (phosphate-buffered saline). The EV preparations derived from virulent Vpb18 and the attenuated variant Apb18 were named, respectively, Vev and Aev.

### 2.3. EV Preparation from Vpb18 Under Sublethal Stress Conditions

We characterized EVs produced by Vpb18 cultivated under sublethal nitrosative (VevNO) and oxidative (VevOxi) stress conditions. The VevNO and VevOxi vesicle samples were prepared from Vpb18 cultivated either in liquid or stationary cultures in Ham/glc medium supplemented with 1.5% glucose incubated for one day at 36 °C, in the presence or absence (control) of stress agents. Liquid cultures were grown under agitation (120 rpm), as detailed previously [7]. VevNO were prepared from Vpb18 cultured at an initial 0.5 mM NaNO_2_ (pH 5.5) concentration and VevOxi were obtained from Vpb18 cultures growing at an initial 5 mM H_2_O_2_ concentration. For EV isolation from liquid cultures, the supernatants were processed as described [7]. Briefly, cell-free supernatants from 500 mL cultures were obtained by two sequential centrifugations of 4000× *g* for 15 min and at 15,000× *g* for 30 min at 4 °C. The supernatants were then filtered through a 0.45-μm cellulose membrane, concentrated to 30 mL in an Amicon system (100,000-Da exclusion limit, Millipore), and ultracentrifuged at 100,000× *g* for 1 h at 4 °C. The pellet was suspended in 300 mL PBS, which corresponded to one EV preparation. EVs from stationary cultures were prepared as described above in Section 2.2 [16]. The stress agent concentration was established following dose–response experiments, in triplicate, on the basis of cell viability and ROS production, as shown in Section 3. The production of ROS, which was indicative of oxidative stress caused in *Paracoccidioides* by both H_2_O_2_ and NaNO_2_, was detected using the fluorescent probe dihydroethidium (DHE), which reacts strongly with superoxide, but also with other oxygen free radicals and NO [23]. Cell pellets were incubated for 30 min at 37 °C with 5 mM DHE (Thermo Fisher, Scientific, Waltham, MA, USA) in fresh Ham/glc, washed 3 times in the same medium and analyzed in a fluorescence microscope (Olympus BX51, Olympus Corporation, Tokyo, Japan) (Olympus System Microscopes). Cell viability was evaluated by incorporation of 0.4% Trypan blue, cell counting in a Neubauer chamber, and also by colony forming units (CFU). For CFU counting, 24-h cultures prepared as described above were diluted and spread (120 µL) in Petri dishes containing mYPD. The number of colonies was counted after 10–15 days of incubation at 36 °C.

### 2.4. Estimation of EV Size, Sterol and Peptide Content

Size and concentration measurements were performed using conventional Nanoparticle Tracking Analysis (NTA), with 3 to 5 captures of 60 s each in a NanoSight NS300 (Malvern Instruments Ltd., Worcestershire, UK) or in a NanoSight LM10 (Malvern Instruments Ltd.). The preparations were diluted to achieve a concentration of 100 particles/frame in sterile PBS (filtered through a 0.22 mm nitrocellulose membrane). The analysis took into consideration the particle diameter (10–1000 nm), the concentration, and the electron density. The images were processed with the aid of the NanoSight Analytical Software-NTA, 3.4 version Build 3.4.4 or earlier (Malvern Instruments Ltd.). The sterol content of EV preparations was estimated using the Amplex^TM^ Red Cholesterol Assay Kit (Invitrogen^TM ^Thermo Fisher Scientific, Waltham, MA, USA) and protein was estimated using the PierceTM BCA Protein Assay (Thermo Scientific). The peptide amount of trypsinized samples for proteome analysis was estimated by spectrometry (A_205_).

### 2.5. Physicochemical Characterization of EVs

Dynamic Light Scattering (DLS) was used to measure the hydrodynamic diameter, polydispersity index (PdI), and surface charge expressed as zeta potential (ZP) in a Zetasizer Nano Zs equipment (Malvern Panalytical Ltd., Malvern, UK). All measurements were performed at 25 °C. For the ZP experiments, the EV suspensions were diluted in 10 mM Hepes, 3 mM NaCl, 1 mM EDTA, pH 7.4, and the measurements were conducted at 150 V. For each sample, three technical replicates consisting of 10 independent fields were taken after a 30-s equilibrium time. For each analytical measurement, at least 3 independently derived EV preparations were analyzed at a concentration of approximately 1010 particles/mL.

### 2.6. Scanning Electron Microscopy (SEM)

SEM analysis was carried out in the Center of Electronic Microscopy, Unifesp. *P. brasiliensis* cells grown for 24 h in liquid cultures containing stressor agents, as detailed above, were pelleted by centrifugation, washed three times in filtered PBS (0.22 mm), and sedimented at 15,000× *g* for 10 min. The cells were fixed overnight o/n in 2.5% glutaraldehyde, 0.1 M sodium cacodylate buffer, pH 7.2 (SC), at room temperature, and processed using the G-O-T-O method [24]. Briefly, fixed samples were washed at room temperature for 30 min and then o/n, fixed in 1% osmium tetroxide in SC for 2 h and washed three times in SC. The samples were then treated with 1% tannic acid in water for 45 min, washed twice in water for 10 min, incubated with 1% osmium tetroxide for 45 min, washed in water 3 times for 10 min, and gradually dehydrated in ethanol in a critical point instrument. The processed samples were mounted in stub, covered with gold by sputtering 25 nm, and analyzed by SEM.

### 2.7. Transmission Electron Microscopy (TEM)

TEM analysis was carried out in the Microscopy Technological Platform of the Carlos Chagas Institute (Fiocruz Paraná, Curitiba, Brazil). For TEM using the conventional negative staining procedure, the EV samples were fixed to formvar-coated grids for 1 h at room temperature, followed by negative staining with 1% uranyl acetate for 10 min. The grids were blotted dry. EVs were observed using a JEOL 1400Plus microscope (Jeol, USA, Peabody, MA, USA) with an acceleration voltage of 100 kV.

### 2.8. Sample Preparation for Proteomic Analysis

Five independent Vev and Aev preparations and three independent VevNO and VevOxi preparations, as well as the non-stressed control grown in Ham/glc, were pooled for proteomic analysis. Protein preparation was carried out in triplicate, as described in Vallejo et al. [7], with modifications. Briefly, the EV samples were dried either in SpeedVac (Vev and Aev) or by lyophilization (VevNO, VevOxi and control) and resuspended in deionized water, and the proteins were precipitated o/n in 10% trichloroacetic acid for 16 h, at −20 °C. Proteins were recovered by centrifugation at 12,000× *g* in a pre-cooled microcentrifuge at 4 °C for 10 min; the pellet was washed once in cold acetone, precipitated in cold acetone for 30 min at −20 °C and pelleted by centrifugation. The samples were denatured at 95 °C for 5 min in methanol/50 mM ammonium bicarbonate (NH_4_HCO_3_) buffer (60:40, *v*/*v*); disulfide bonds were reduced by treatment with 5 mM dithiothreitol (DTT) for 30 min at 37 °C; cysteine residues were alkylated upon incubation for 90 min at room temperature, in the dark, with iodoacetamide to a final concentration of 10 mM. Protein digestion was performed o/n with one μg of recombinant sequencing-grade trypsin (Promega), at 37 °C, and the reaction was terminated by addition of 0.05% trifluoroacetic acid.

For proteome sequencing of Vpb18 and Apb18 yeast cells, the proteins were extracted from *P. brasiliensis*, according to Castilho et al. [20], with modifications. Yeast cells cultivated as for EV preparation were recovered by centrifugation at 2000× *g* for 15 min, at 4 °C, and washed three times in cold PBS. The washed pellet was resuspended in 50 mM ammonium bicarbonate (pH 8.5) and mechanically disrupted by vortexing with glass beads (5 cycles of 1 min, 1 min-interval in ice). The lysate was centrifuged at 2500× *g* for 5 min at 4 °C and the glass bead-free supernatant was subjected to sonication (4 pulses of 30 s, 40 Hz, with 1 min-intervals in ice). The supernatant was collected by centrifugation at 15,000× *g* for 10 min, at 4 °C. The protein concentration was estimated using the Pierce^TM^ BCA Protein Assay Kit (Thermo Scientific), 150 μg of protein were reduced with 5 mM DTT for 30 min, at 65 °C, and alkylated with 15 mM iodoacetamide for 1 h at room temperature, in the dark. The proteins were precipitated with 9 volumes of cold acetone and methanol solution (8:1) and dried in a vacuum concentrator. The proteins were subsequently dissolved in 5 μL of 100 mM NaOH and digested with 2 μg of sequencing-grade modified trypsin (Promega Corporation, Madison, WI, USA) in 195 μL of 50 mM HEPES buffer (pH 7.5) for 16 h, at 37 °C.

The resulting peptides were desalted in a POROS R2 column (Applied Biosystems, , Foster City, CA, USA), the peptides were eluted with 80% acetonitrile in 0.1% trifluoroacetic acid and dried in a vacuum concentrator. The samples were stored at −20 °C prior to nano LC-ESI-MS/MS analysis.

### 2.9. LC-MS/MS Analysis

The resulting tryptic peptides from Vev, Aev, Vpb18, and Apb18 were separated using nano-flow liquid chromatography (UltiMate^TM^ 3000/Thermo Fisher Scientific, Sunnyvale, CA, USA). A total of 1.5 μg peptides were loaded onto a first-dimension column Acclaim PepMap RSLC C18 (75 µm × 150 mm, 2 µm, 100 Å pore, nanoViper). The mobile phase consisted of (A) 0.08% formic acid in HPLC-grade H2O and (B) 80% acetonitrile, 0.08% formic acid. Peptides were eluted in a 2–40% gradient of buffer B over 93 min, at a flow rate of 350 nl/min, and injected for mass spectrometry (MS) analysis. The source was operated in nano-ESI (+) positive ionization mode. MS analysis was performed in an Impact II (Bruker Daltonics, Billerica, MA, USA) equipped with a NanoElectronSpray source and two mass analyzers: a quadrupole and a time-of-flight (TOF) sensor operating in V-mode. For VevNO, VevOxi, and control samples, chromatography of equivalent peptide amounts of each sample was carried out in a nanoAquity UPLC (Waters Corporation, Milford, MA, USA), coupled to a mass spectrometer Synapt G2 HDMS (Waters), in the presence of known amounts of a peptide marker. Samples dissolved in 0.1% formic acid were injected in a trap column (C18 nanoAcquity trap Symetry, 180 mm × 20 mm, Waters) and the peptides were eluted in an analytical capillary column (C18 nanoAquity BEH 75 mm × 150 mm, 1.7 mm) using a gradient of 7% to 35% B (99.9% acetonitrile, 0.1% formic acid) up to 35% B for 92 min at 275 nL/min. Data acquisition in the HDMSE mode was performed by alternating the low (4 eV) and high collision energy modes with ramps of 19 to 45 eV each 1.25 s. The mass spectrometer was calibrated before and during the analytical run at 60 s intervals using the nanoLockSpray source with the GFP-b peptide (glufibrinopeptide, Waters). Biological replicates were analyzed in technical triplicates.

### 2.10. Proteomic Data Processing

The proteomic data files were processed using PEAKS Studio 8.5 (Bioinformatics Solutions Inc., Waterloo, ON, Canada) and the Proteome Discoverer Suite version 2.4 (Thermo Scientific, Waltham, MA, USA). The processing parameters of MS/MS spectra were set as follows: parent mass error tolerance at 0.07 Da; fragment mass error tolerance at 0.07 Da; strict trypsin specificity, allowing three missed cleavages; minimum peptide length of five amino acids; detection of at least two fragment ions per peptide; max variable PTM per peptide set to three; carbamidomethylation of cysteine as a fixed modification, and oxidation of methionine as variable modifications. To ensure high confidence, a false discovery rate (FDR) threshold of 1% was applied on the peptide-spectrum match (PSM). The search was conducted against the *P. brasiliensis* strain Pb18 database UP000001628 (8399 entries, https://www.uniprot.org/proteomes/UP000001628 (accessed on 27 September 2023)). Proteins detected with two or more peptides, one of which being unique, were validated if present in at least two out of three technical replicate injections. Proteins identified with low accuracy were excluded. Mass spectrometry data have been deposited to the ProteomeXchange Consortium via the MassIVE (MSV000098328) partner repository with the dataset identifier: PXD065468.

### 2.11. Protein Abundance Analysis, Functional Annotation, and PTM Profiling

The proteomic data were annotated as log2, and differential protein abundance analysis was performed using the R/Bioconductor package Limma (version 3.60.4). Differences in proteome profiling between the samples were identified using the empirical Bayes moderated t-statistics test, with Benjamini–Hochberg corrections applied to all *p*-values to calculate false discovery rates (FDR). Proteins were considered differentially abundant (DAPs) between two groups if they met the following criteria: *p* value < 0.05 and log2 fold change < −0.38 or >0.38. Canonical pathway enrichment analysis was performed using STRING database (version 12.0; https://string-db.org/, accessed on 26 February 2025). All validated proteins identified in each sample were included in the analysis. Initially, these proteins were annotated into Gene Ontology (GO) categories—including Molecular Function, Cellular Component, and Biological Process—as well as Kyoto Encyclopedia of Genes and Genomes (KEGG) and Reactome pathways. For each annotated term, the percentage of proteins in the query list relative to the total number of validated proteins was calculated and referred to as % query. Next, the percentage of proteins associated with the same functional categories across the entire genome was calculated relative to the total number of proteins encoded by the genome (% universe). Fold enrichment for each GO, KEGG, or Reactome category was then calculated by dividing % query by % universe, representing the degree of overrepresentation of each category in the sample compared to the genomic background. In a second step, each list of higher-abundance proteins was analyzed separately to identify the most significantly enriched pathways and their predicted states. A false discovery rate (FDR) of 0.05 or lower was used as the significance threshold. Finally, a protein–protein interaction network (PPIN) was generated using the list of unique and higher-abundance proteins in the Vev and Aev samples. Interactions were obtained from the STRING database (© STRING Consortium 2024, STRING v12.0), using a confidence score cutoff of 0.4 (medium confidence) and a false discovery rate-corrected *p* value < 0.05, with no additional interactors added. The identification of protein post-translational modifications (PTMs) was accomplished using the PTM profile tool to define the presence and position of PTM sites.

## 3. Results

In order to study the differences between extracellular vesicles released by virulent *P. brasiliensis* Vpb18 (Vev) yeast cells and its attenuated Apb18 form (Aev), we compared their physicochemical properties and proteomes. We also evaluated these parameters for Vev from Vpb18 yeast cells cultivated under sublethal oxidative (VevOxi) and nitrosative (VevNO) stress for comparison.

### 3.1. Vev and Aev Morphological and Physicochemical Aspects

Vev and Aev were isolated by differential filtration and centrifugation of washes from stationary Vpb18 and Apb18 yeast culture in defined Ham/glc medium [16]. The EV pellets, obtained by a final ultracentrifugation at 100,000× *g* for 1 h, were suspended in PBS and analyzed immediately after or within a 7-day-period storage at 4 °C.

**Figure 1:**
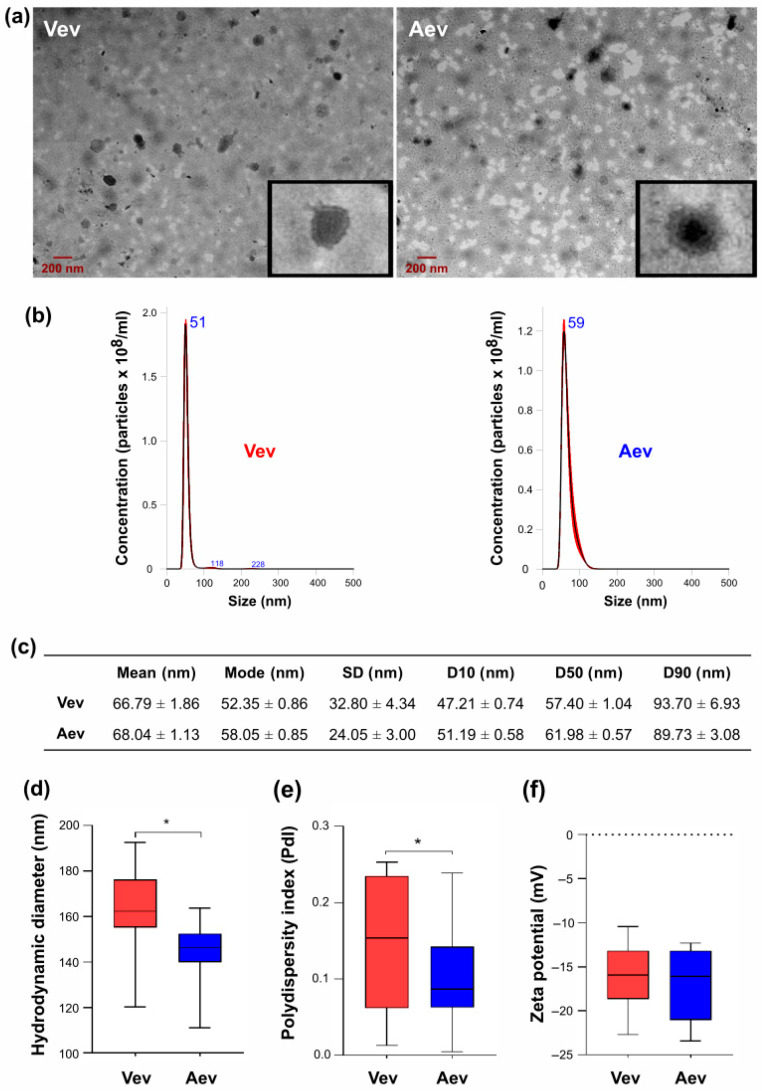
Morphological and physicochemical properties of Vev and Aev from the yeast *P. brasiliensis* form of virulent Vpb18 and attenuated Apb18. (**a**) TEM images of Vev and Aev negative staining. A 200 nm-size bar is indicated. The insets show an amplified EV particle in each sample. (**b**) NTA histograms of representative Vev and Aev preparations (5 captures of 60 s each) showing particle concentration distributions according to the size in nm. The peak sizes are indicated. (**c**) NTA size distribution of Vev and Aev calculated from 10 independent preparations. The D10, D50, and D90 parameters indicate the diameters below which 10%, 50%, and 90% of the EV population fit, respectively. (**d**–**f**) Vev and Aev (**d**) hydrodynamic diameters (DLS) in nm, (**e**) polydispersity index (PI), and (**f**) zeta potential. Values were expressed as the median of three measurements per replicate. *, *p* < 0.05 was considered statistically significant.

The images obtained by negative staining (Figure 1a) suggested the presence of purified vesicles in both Vev and Aev preparations. However, the populations exhibited a heterogeneous profile in terms of morphology, diameter, and electron density. Most EVs appeared spherical or ovoid, with a lipid bilayer, similar to those described for other fungal pathogens [25,26,27]. The EV electron densities varied considerably, suggesting distinct contents [28]. The diameters were generally below 200 nm and the surface appeared rough, as shown in the inset (Figure 1a).

The physicochemical properties, especially those associated with surface charge, can potentially affect extracellular vesicle transport and specific interactions, thus interfering with cell–cell communication [29]. In our experimental conditions, the mean diameter estimated by NTA (Figure 1b,c) was similar for Vev (66.79 nm) and Aev (68.04 nm), with values resembling those from our previous publication [16]. However, we observed that the DLS hydrodynamic median diameters were higher than NTA values (Figure 1d). DLS values indicate the degree of dispersion caused by the Brownian movements of the particles in suspension and reflect the EV surface interaction with the medium, including a solvent layer and adsorbed aggregates [30]. The presence of different membrane constituents can affect this interaction. The DLS median value was statistically higher for Vev (163.06 nm) than for Aev (143.71 nm), suggesting distinct surface characteristics (Figure 1d). The polydispersity index (PdI) was also statistically higher for Vev (0.14) than for Aev (0.10) (Figure 1e), suggesting that the Aev particle size distribution is more homogeneous. The zeta potential (ZP) serves as an indicator of colloidal stability and can be used to predict EV aggregation or internalization by target cells [31]. The ZP is modulated by the amount and distribution of phospholipids, carbohydrates, associated nucleic acids, and proteins in the EV membrane [32]. We observed similar ZP median values (−16.20 ± 3.78 mV and −17.06 ± 4.18 mV) for freshly isolated Vev and Aev (Figure 1f), suggesting that differences in surface characteristics do not affect their net charge under our experimental conditions. Notably, we previously observed that Vev, but not Aev, tended to aggregate when stored in PBS at −20 °C and 4 °C for over 15 days [16], reinforcing the fact that these vesicles bear different surface characteristics.

### 3.2. P. brasiliensis Vev and Aev Proteomes Differ in Cargo Profile

In order to understand the differences between the Vev and Aev proteomes, the proteins were precipitated, trypsin-digested, and the resulting peptides were analyzed by nano LC-ESI-MS/MS. Abundance of each protein was estimated by label-free quantification and compared as peak area values. We also analyzed the proteome of total extracts from the Vpb18 and Apb18 parent yeast cells in order to make a direct correlation with the respective vesicle proteins.

**Figure 2:**
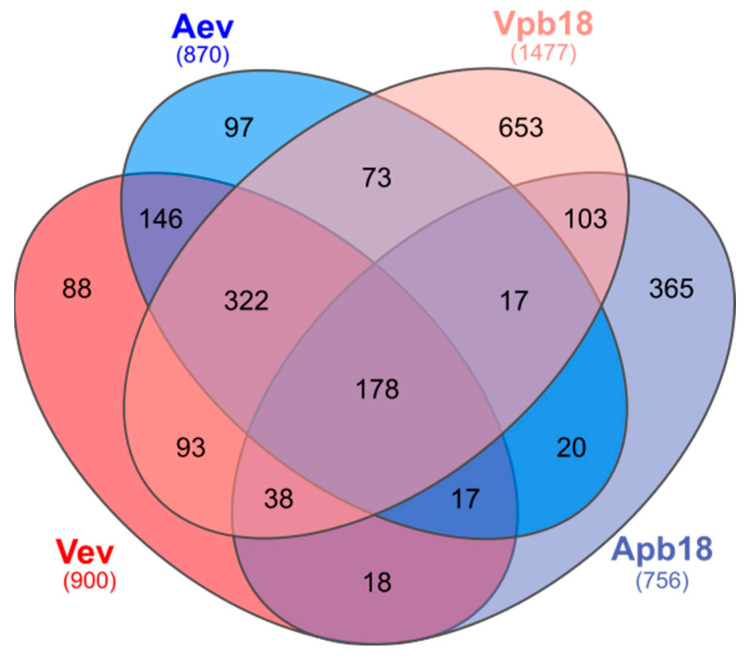
Venn diagram showing the number of validated proteins identified through nano LC-ESI-MS/MS in Vev, Aev, and the corresponding parent cell extracts (Vpb18 and Apb18). The diagram shows the number of overlapping proteins, while the total number in each sample is indicated in parenthesis.

The proteome raw data resulted in 12,056 peptide sequences corresponding to 1282 proteins identified in Vev, as compared to 11,158 peptides and 1264 proteins in Aev. Proteomic analysis of the respective cell extracts identified 14,729 Vpb18 and 5712 Apb18 peptide sequences, corresponding to 1979 Vpb18 and 1289 Apb18 proteins. For further analysis, we considered only proteins validated under the strict parameters detailed in Materials and Methods, which resulted in 900 Vev, 870 Aev, 1477 Vpb18, and 756 Apb18 sequences (Figure 2). We cannot determine whether the lower number of peptides detected in the Apb18 extract was due to reduced protein expression or to technical limitations, as the detection of low-abundance proteins within complex mixtures may be hindered by analytical sensitivity constraints. Therefore, the results for Apb18 were used with care or not considered during further analysis, especially because the Vpb18 and Apb18 proteomes were numerically similar in a previous publication [20].

A total of 234 Vev and 243 Aev unique sequences were not among the validated proteins in the cell extracts (Figure 2), probably because they were present at low amounts in the cell extracts. If so, it is relevant that they were concentrated in EVs. Additionally, since the unique EV sequences include 118 transmembrane proteins, we cannot disregard the possibility that some were not properly extracted from the *Paracoccidioides* yeasts cells. The complete dataset is available in the ProteomeXchange repository PXD065468. We should mention that although the total number of transmembrane proteins was the same in Vev and Aev (143) samples, 41 were differently abundant, which may explain the differences in DLS measurements described in the previous section.

It is noteworthy that the most abundant sequences were similar in the Vev and Aev samples. The plasma membrane ATPase (C1GM00), catalase (C1G0D4), and moonlight glyceraldehyde-3-phosphate dehydrogenase (C1G5F6) were on top of the list, but we also highlight elongation factor 1-alpha (C1G1F2), heat shock Hsp90 (C1GKC9), and Hsp72 (C1GLI2), GTP-binding nuclear protein (C1GCT8), and amidase (C1GHS5). These proteins were also abundantly present in the Vpb18 proteome.

**Figure 3:**
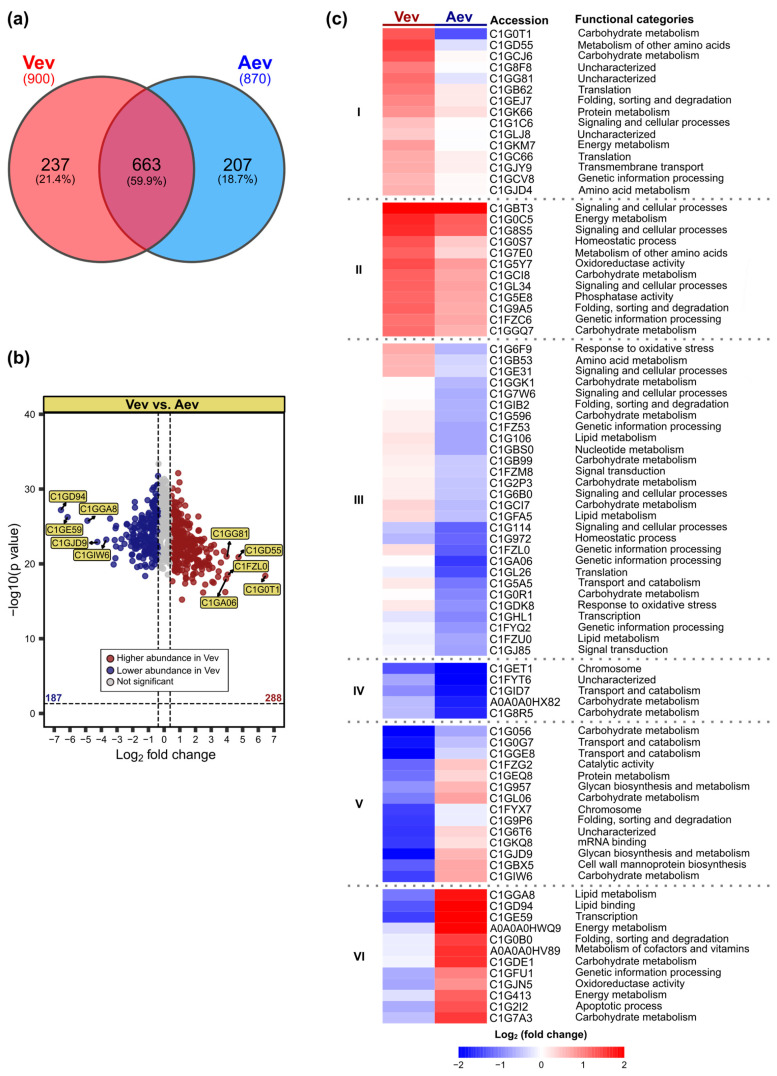
Analysis of Vev and Aev overlapping proteins. (**a**) Venn diagram showing the number (and percentages) of overlapping and unique proteins in Vev and Aev. (**b**) Volcano plot distribution of overlapping proteins with higher (288, red dots) or lower (187, blue dots) abundance in Vev, compared to Aev, out of 475 differentially abundant proteins (DAPs) with *p* < 0.05 and log_2_FC ≤ −0.38 or ≥0.38. We indicated the top 5 proteins with higher or lower abundance, which are glycogenin-1 (C1G0T1), 5-oxoprolinase (C1GD55), T-complex protein 1 subunit delta (C1FZL0), PHD-type domain-containing protein (C1GG81), peptidyl-prolyl cis-trans isomerase (C1GA06), 6PF2K domain-containing protein (C1GIW6), dolichyl-diphosphooligosaccharide-protein glycosyltransferase subunit WBP1 (C1GJD9), AMP-binding domain-containing protein (C1GGA8), RNA helicase (C1GE59), and bactericidal permeability-increasing protein (C1GD94). Gray dots, proteins with statistically similar abundance in both samples. (**c**) Heatmap of 86 DAPs (red, higher abundance; blue, lower abundance) in either Vev or Aev, considering at least 4-fold differences (log_2_FC ≤ −2 or ≥2 and *p* < 0.05). Clustering (I–VI) was based on similar abundance trends across samples. The primary functional GO category of each protein is indicated.

When comparing the vesicle proteomes (Figure 3), 237 sequences were unique to Vev and 207 were unique to Aev (Figure 3a). Out of 663 overlapping proteins, 288 (43%) were significantly enriched in Vev, while 187 (28%) were enriched in Aev (Figure 3b).

The heatmap in Figure 3c shows 86 proteins that were at least 4-fold differentially abundant in either Vev or Aev (*p* < 0.05 and log_2_FC ≤ −2 or ≥2). Of these, 60 were enriched in Vev (I to IV), while 24 were more abundant in Aev (V and VI). The higher abundant proteins pointed in the volcano plot of Figure 3b are in clusters I and III, while the lower abundant sequences are in clusters V and VI (Figure 3c). According to the GO primary biological process of each protein, response to oxidative stress and notably signaling and cellular processes are characteristic of clusters I to III. Proteins that were enriched 15 to 87-fold in Vev are directly or indirectly linked to the fungal resistance processes, including glycogenin-1 (C1G0T1), peptidyl-prolyl cis-trans isomerase (C1GA06), and 5-oxoprolinase (C1GD55).

**Figure 4:**
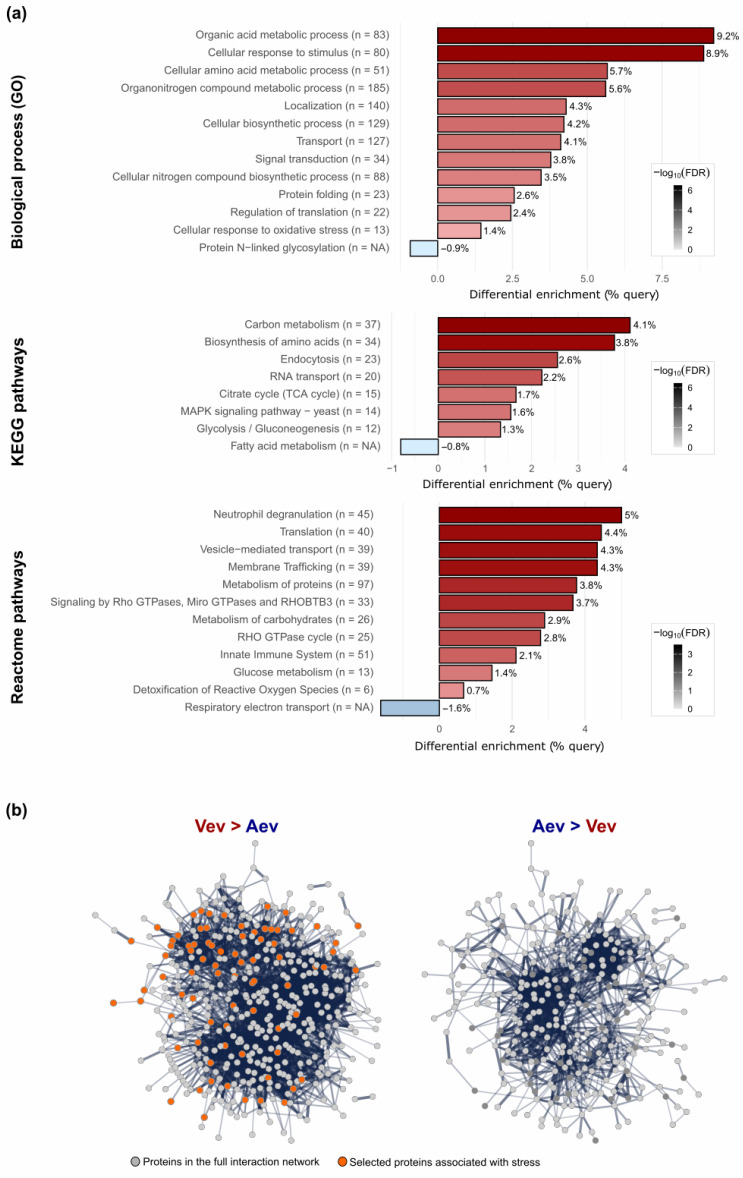
Functional enrichment analysis of unique/higher abundance Vev proteins relative to Aev. (**a**) Proteins were categorized according to Gene Ontology (GO) biological processes, KEGG and Reactome pathways. For each selected term, we show the differential percentage of Vev proteins (compared to Aev) in the query list relative to the total number of validated proteins. The plot displays both the “% query” and the number of proteins associated with each category. Enrichment was determined using STRING-db functional enrichment analysis with an FDR cutoff of 0.05. The color intensity scale represents the –log_10_(FDR). The terms were selected on the basis of biological relevance, functional diversity, and broad coverage. (**b**) Protein–protein interaction (PPI) network of unique/higher abundance proteins in Vev (Vev > Aev) and Aev (Aev > Vev). Multiple lines represent a higher number of interactions, while a single line indicates one interaction. Gray dots, proteins present in the full PPI network; orange dots, proteins associated with response to general stimulus, cellular response to stress, detoxification of reactive oxygen species, and related terms, according to GO, KEGG, and Reactome annotations. Disconnected nodes were removed to enhance clarity.

An enrichment analysis of unique/enriched proteins in Vev (527 sequences) and Aev (497 sequences) was performed using STRING (version 12.0). Functional enrichment was assessed based on the false discovery rate (FDR), corresponding to adjusted *p*-values. The analysis resulted in the following number of significantly enriched terms: for GO biological processes, 174 in Vev and 63 in Aev; for KEGG pathways, 26 in Vev and 6 in Aev; for Reactome pathways, 36 in Vev and 12 in Aev. We found 36 GO terms related to fungal virulence exclusively enriched in the Vev proteome. Some of them are represented in Figure 4a, such as response to oxidative stress (FDR = 0.0042), signal transduction (FDR = 0.0127), transport (FDR = 1.32 × 10^−8^), localization (FDR = 1.86 × 10^−9^), and regulation of translation (FDR = 0.0057). Terms within the KEGG and Reactome pathways also suggest the predominance of virulence factors in Vev over Aev, such as MAPK signaling (FDR = 6.60 × 10^−4^), signaling by GTPases (FDR = 6.46 × 10^−5^), translation (FDR = 5.65 × 10^−5^), and detoxification of reactive oxygen species (FDR = 0.0444). Moreover, Reactome terms like neutrophil degranulation (FDR = 3.17 × 10^−6^) and innate immune system (FDR = 1.72 × 10^−5^) indicate the potential interaction of a number of Vev higher abundant/unique proteins with the host immune system (Figure 4a). The differential functional enrichment between Vev and Aev unique/enriched proteins is well reflected in the striking difference seen in the protein–protein interaction network (Figure 4b), which shows a higher number of edges for Vev. Also notable is the presence of proteins associated with stress response only in the Vev PPI graphic (orange dots). It is worth mentioning that functional differences observed between the Vev and Aev enriched/unique sequences were also observed when the Vev and Aev proteomes were compared with the *P. brasiliensis* strain Pb18 genome (Figure S1). The Vev, and not Aev, proteins were more enriched than the Pb18 genome within the terms of cellular response to oxidative stress (GO:0034599) (FDR = 0.0033) and cell wall chitin metabolism (GO:0006038) (FDR = 0.0070), which are related to virulence.

The data presented so far shows that the Vev, the Aev, and the Vpb18 proteomes differ, suggesting there is selective bias in the Vev protein cargo. The differences in protein load are translated into differences in functional enrichment terms and protein–protein interaction.

### 3.3. Protein Nt-Acetylation and Phosphorylation Vary with the Sample

Post-translational modifications (PTMs) are essential mechanisms developed by eukaryotic cells to enhance protein diversity [33]. Therefore, we broadened our proteome analysis using PEAKS PTM (BSI PEAKS), which is a de novo-based database search engine optimized for PTM detection. When all PTMs were considered, the number and type were generally similar between Vev and Aev sequences (repository PXD065468). We then deepened our analysis on *N*-terminal acetylation (Nt-Ac) and phosphorylation (Pho), considering that Nt Ac can interfere with protein enzymatic activity, stability, folding, interactions, cellular localization [34], and phosphorylation plays critical roles in a number of cellular processes, including cell cycle regulation, growth, and signal transduction [35]. It is of note that although our samples were not specifically processed to preserve PTM, the detection of Nt-Ac is considered reliable due to the stable and irreversible nature of this covalent modification, which is resistant to standard sample preparation protocols [36]. The protein phosphorylation data, on the other hand, reflect only the most stable or highly abundant sites [37]. Therefore, they were only used as a preliminary source of comparison.

**Figure 5:**
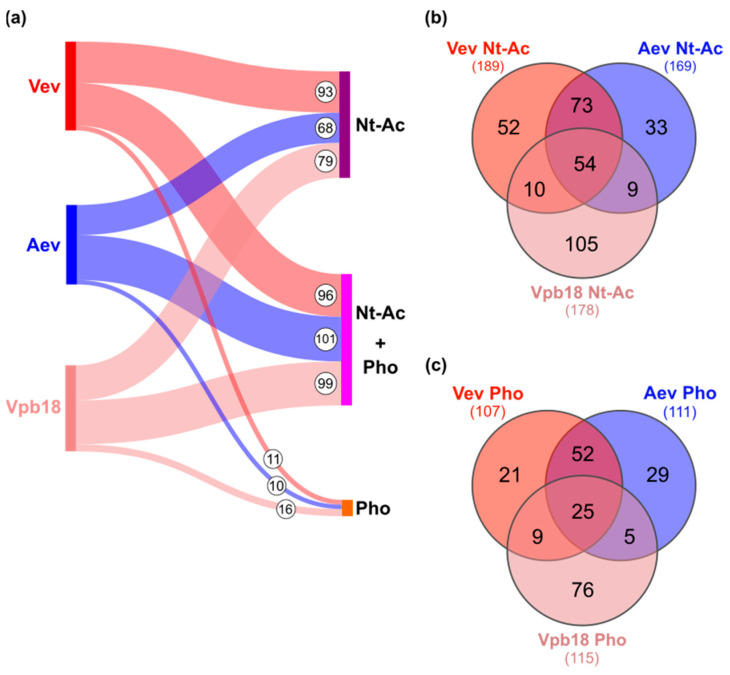
*N*-terminal acetylation (Nt-Ac) and phosphorylation (Pho) in Vev, Aev and Vpb18 proteins. (**a**) Sankey diagram showing the number of Nt-acetylated proteins and Pho at serine, threonine, or tyrosine residues identified in each sample. (**b**) Venn diagram summarizing the number of shared Nt-acetylated proteins across the three proteomes indicated. (**c**) Venn diagram summarizing the number of shared phosphorylated proteins across the three proteomes indicated.

The Sankey diagram in Figure 5a shows that out of 189 proteins bearing Nt-Ac in Vev, 96 also included Pho (50%). The proportion of Nt-Ac was higher in Vev (21%) than Aev (19%) and, especially, than Vpb18 (12%) proteins, suggesting that there is a trend for Nt-Ac in vesicle proteins. This trend was also observed for Pho: 12% in Vev, 12.75% in Aev, and 7.8% in Vpb18.

The interesting observation about the status of Nt-Ac and Pho is that it varied with the sample for the same protein, suggesting the existence of the dynamic modulation of protein modification in extracellular vesicles. Figure 5a shows that 52 Nt-acetylated proteins in Vev are not Nt-acetylated or not present in the other samples, while 54 proteins are Nt-acetylated in all three samples. Figure 5c reveals that 25 proteins display Pho in all samples. Therefore, we found that the presence of Nt-Ac in the same protein depended on the sample analyzed (Vev, Aev, or Vpb18), strongly suggesting that Nt-Ac may have a role in extracellular vesicle localization and/or protein function. Specific examples will be further explored in the next sessions.

### 3.4. Sublethal Stress Conditions Induce Altered Vev Proteome

In order to evaluate how oxidative and nitrosative stress culture conditions can affect the *P. brasiliensis* EV characteristics and correlate stress with unique/enriched Vev proteins, Vpb18 was cultivated at sublethal concentrations of the stress agents H_2_O_2_ and NaNO_2_. We chose to work at sublethal concentrations to keep cell viability above 95%, therefore avoiding artifacts in the EV preparations due to cell debris-derived membranes.

**Figure 6:**
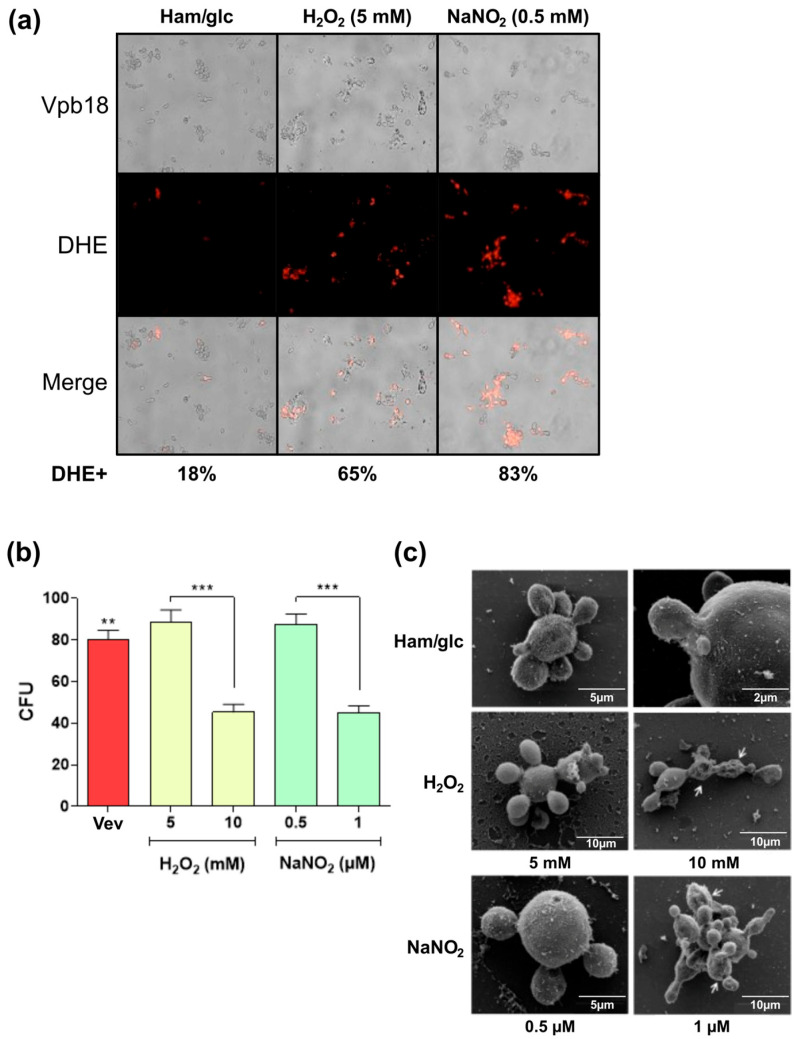
Effect of oxidative (H_2_O_2_) and nitrositive (NaNO_2_) sublethal stress on *P. brasiliensis* Vpb18 yeasts. (**a**) DHE fluorescence detection of ROS (red spots) by direct microscopy of Vpb18 yeast cells treated with the indicated stress-inducing agents and initial concentrations. The values represent the mean percentages of DHE+ cells (DHE staining) from three independent replicates. (**b**) Colony forming units (CFU) after 24 h of fungal growth in Ham/glc medium alone (ctr) or in the presence of the indicated agents and concentrations (** *p* < 0.002; *** *p* < 0.0004). (**c**) Scanning electron microscopy (SEM) of Vpb18 yeast cells treated with the indicated stress agents and initial concentrations. The arrows point to the areas of morphological changes in the cells and the bars indicate the size in mm.

Dose–response experiments revealed that Vpb18 cultivated for one day at 5 mM H_2_O_2_ and 0.5 mM NaNO_2_ initial concentrations had similar cell viability and morphology to the control (Figure 6). However, they were under stress, considering that 65% (H_2_O_2_) and 81% (NaNO_2_) of the cells emitted red fluorescence due to DHE oxidation (Figure 6a). Concentrations of 10 mM H_2_O_2_ and 1 mM NaNO_2_ also prompted ROS production, but with statistically significant reduction in cell viability (Figure 6b) and visible cell damage (Figure 6c). Therefore, we proceeded with proteomics of vesicles produced by one-day Vpb18 cultures in the presence of 5 mM H_2_O_2_ or 0.5 mM NaNO_2_ initial concentrations, because these conditions caused sublethal stress in both liquid and stationary cultures.

The EVs produced under sublethal stress conditions were called VevOxi (oxidative) and VevNO (nitrosative). TEM analysis of these samples revealed the presence of spherical structures with electron-dense membrane bilayers characteristic of EVs (Figure S2a). The mean NTA diameters estimated for VevOxi and VevNO were 61 nm and 79 nm (Figure S2b), respectively, statistically similar to the controls (62 nm). The mean DLS diameter was also similar for VevNO and its lower pH control (172 nm and 181 nm, respectively), however significantly higher for VevOxi (213 nm) than the control (160 nm). The polydispersity index was also statistically higher for VevOxi (0.45 vs. 0.37), but not for VevNO, suggesting that VevOxi are more heterogeneous than the control. The ZP values were similar among the samples (between −18.8 and −22.1). Sterol and protein contents were similar for VevNO and the control, but sterol was statistically lower for VevOxi.

To compare the chemical composition of the EV surfaces, we have also used a variant of Raman spectroscopy called surface-enhanced Raman Scattering (SERS) [38]. Raman spectroscopy is a label-free vibrational technique that provides information at molecular level about the composition of biological samples including protein, lipids, and nucleic acids [39]. In the case of SERS, the Raman signal is highly enhanced, allowing sensitive information about the molecular composition of the samples, including extracellular vesicles [40]. Preliminary assays were carried out using silver nanoparticles synthetized by the hydroxylamine method. A 785 nm excitation laser showed that the SERS spectra from VevOxi and VevNO samples shared similar vibrational profiles, which were generally distinct from the Vev profile, thus suggesting overall differences in the particles deriving from stressed *Paracoccidioides*. The spectral profiles obtained for Vev and Aev, on the other hand, were quite similar.

Together, our data suggest that sublethal oxidative stress culture conditions resulted in altered VevOxi surface composition and decreased sterol amounts; however, the net surface charge was not affected. In contrast, sublethal nitrosative stress did not affect the VevNO features. It is worth mentioning that we observed an increased yield of vesicles isolated from stressed Vpb18 cultures, which aligns with the literature data [41].

**Figure 7:**
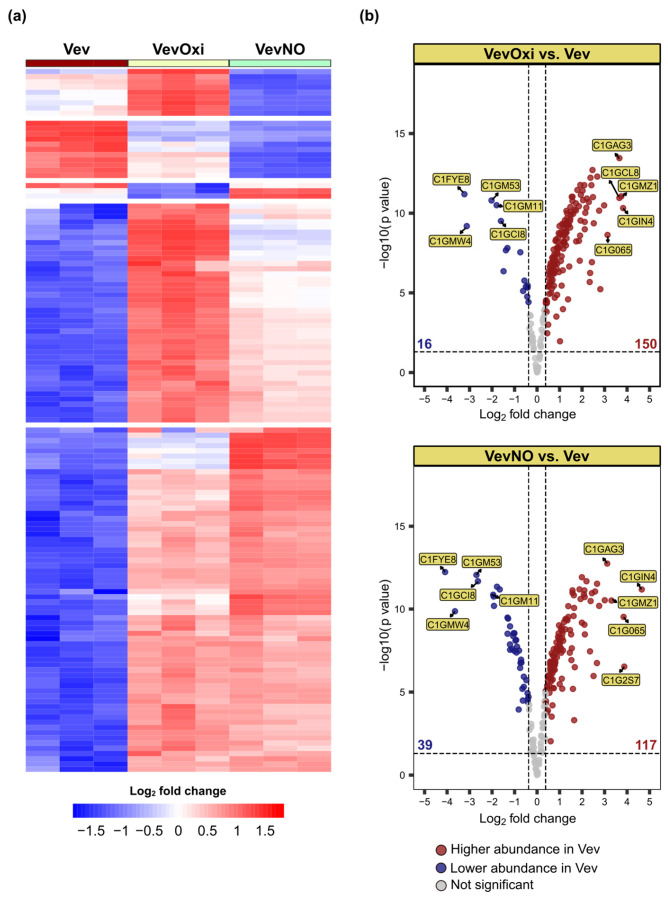
Analysis of the VevOxi and VevNO proteomes from extracellular vesicles produced under sublethal oxidative and nitrosative stress conditions. (**a**) Heatmap showing the relative abundance of proteins identified in each biological replicate (columns) of Vev (control), VevOxi, and VevNO, as indicated. The color scale indicates abundance (blue, lower abundance; red, higher abundance). (**b**) Volcano plot of differentially abundant proteins (DAPs) in VevOxi and VevNO. DAPs were identified based on *p* < 0.05 and log2FC ≤ −0.38 or ≥0.38, relative to the Vev control. Red dots, increased abundance; blue dots, decreased abundance; gray dots, proteins with statistically similar abundance in both samples. We indicated the top 5 proteins with higher or lower abundance, which are: annexin (C1GIN4), peroxisomal hydratase-dehydrogenase-epimerase (C1GMZ1), isocitrate dehydrogenase [NADP] (C1GAG3), malonyl-CoA transacylase (MAT) domain-containing protein (C1G065), catalase (C1GCL8), Rab GDP dissociation inhibitor (C1G2S7), nucleotidyl transferase AbiEii/AbiGii toxin family protein (C1GM11), tropomyosin (C1GMW4), citrate synthase (C1GC18), antigenic thaumatin-like protein (C1GM53), hyaluronan/mRNA-binding protein domain-containing protein (C1FYE8) The numbers indicate the total count of DAPs in each comparison.

The proteomic analysis of VevOxi and VevNO samples produced under sublethal stress resulted in the identification of 376 proteins in VevOxi, 376 in VevNO, and 369 in Vev. Of these, 213 sequences, all common across samples, were validated as defined by their presence in at least two replicates per sample, *p* < 0.05, and a minimum of two identified peptides, with one being unique (Figure 7). These sequences were obtained at lower sensitivity conditions some years previously to the Vev/Aev proteome sequencing and originated from EV produced by one-day cultures, thus justifying the comparative lower protein sequence numbers.

In the heat map shown in Figure 7a, it is clear that most of the 213 validated proteins are more abundant in the VevOxi and VevNO samples than in the control. The Volcano plot in Figure 7b shows that 150 VevOxi and 117 VevNO sequences were significantly enriched, while only 16 VevOxi and 39 VevNO proteins were less abundant. Unique proteins were not detected. We found a number of stress-related proteins enriched in VevOxi and VevNO, maybe following an anticipatory stress response under mild stress conditions [42]. The highest-abundant sequences were annexin (C1GIN4), peroxisomal hydratase-dehydrogenase-epimerase (C1GMZ1), isocitrate dehydrogenase [NADP] (C1GAG3), and malonyl-CoA transacylase (MAT) domain-containing protein (C1G065), catalase (C1GCL8), and Rab GDP dissociation inhibitor (C1G2S7). All of them are related to stress adaptation and some, like catalase, can protect the cell extracellularly. Functional analysis of GO biological process, KEGG and Reactome pathways for VevOxi and VevNO enriched proteins, compared to the fungal genome, revealed enrichment in pathways related to the response to toxic substances, oxidative stress, cellular detoxication, glutathione metabolism, detoxification of reactive oxygen species, regulation of protein kinase (PKN) activity by Rho GTPases, neutrophil degranulation, and innate immune responses (Figure S3).

We then correlated the proteins that were significantly enriched in VevOxi and VevNO with those significantly enriched/unique in Vev relative to Aev (Table 1). This analysis is relevant because it points to Vev-enriched proteins whose abundance increases when Vpb18 is under sublethal oxidative and nitrosative stress conditions.

The analysis resulted in 70 enriched VevOxi and/or VevNO proteins that were also enriched/unique in Vev vs. Aev (Table 1). In general, these proteins are associated with anti-oxidation, transport, heat stress, and cell wall/carbohydrate metabolism. Of these, 54 proteins were enriched in both VevOxi and VevNO, but for several of them the abundance is visibly higher in one of the samples. This is true for GAPDH (C1G5F6), superoxide dismutase (C1G4T8), enoyl-CoA hydratase (C1G2P3), and citrate synthase (C1GLZ6), which are prevalent in VevOxi produced under sublethal oxidative stress, and for glycosidase (C1G8V5), which is prevalent in VevNO.

A number of fungal molecules have been reported as virulence-associated, favoring the process of fungal infection, survival, pathogenicity, as well as the modulation of host defenses through mechanisms such as immune evasion, oxidative stress resistance, and interference with host signaling pathways [43]. We then searched for fungal virulence factors and regulators reported in the literature [13,44,45,46,47,48,49] and data bank descriptions that were present in the proteomes characterized in this work. In total, we found 194 virulence-associated accession numbers and they represent 47% of the proteins in Table 1. In counterpart, only 27% of the virulence factors enriched in VevOxi and/or VevNO are not enriched in Vev relative to Aev (Table S1). Among them are two catalases (C1GCL8, C1G0D4), peroxidase (C1G7K8), rab GDP dissociation inhibitor (C1G2S7), two GTP-binding proteins (C1GLV1, C1GM08), alpha-glucan synthase (C1G7L4), two 14-3-3-associated proteins (C1GB04, C1G9X0), and two aminopeptidases (C1GA81, C1GA81), which are enriched in Vev due to sublethal stress, but not relative to Aev.

### 3.5. Vev-Enriched Fungal Virulence-Associated Proteins Tend to Be Nt-Acetylated and More Abundant Due to Sublethal Stress

We identified 16% (145 accession numbers) virulence-associated proteins in the Vev and 14% (124 accession numbers) in the Aev proteomes. Although the percentages were similar, 64% potential virulence factors were either unique (27) or significantly enriched (66) in Vev relative to Aev. It is notable that 10% of the identified virulence-associated proteins were found in the Vpb18 yeast proteome, but only 33 out of 149 proteins (three bearing Nt-Ac) were unique (repository PXD065468)). Therefore, about 78% of the Vpb18 potential virulence factors were delivered in vesicles, of which 57% are enriched in Vev relative to Aev, especially in GO groups identified as antioxidants, heat shock, transport, and signaling proteins (Table 1 and Table 2). Consequently, we focused on the analysis of Vev-enriched virulence factors, taking into account their fold-change enrichment in Vev (Vev > Aev), VevOxi/VevNO from stressed *P. brasiliensis* (HAP), and considering the presence of Nt-Ac comparatively in the Vev, Aev, and Vpb18 samples (Table 2). Differential Nt-Ac has particularly attracted our attention. Considering all proteomes, 68 virulence-associated proteins were Nt-acetylated (repository PXD065468)): 22 in all samples; 18 in Vev, not in Aev; 22 in Vev, not in Vpb18 (where 7 proteins were absent); 15 in Vpb18, not in Vev. In Table 2, we observed that 32% of the Vev-enriched virulence factors showed Nt-Ac, but it varied with the sample without following a specific pattern. Phosphorylation was detected in only 19 Vev and 11 Aev proteins and was not further analyzed (repository PXD065468)).

In Table 2, Vev-enriched enzymes with primary function in carbohydrate metabolism were generally enriched upon sublethal stress as well. Their role in virulence is mostly due to secondary functions as moonlight proteins [49]. For instance, glyceraldehyde-3-phosphate dehydrogenase (C1G5F6) and triosephosphate isomerase (C1G120), traditionally involved in energy metabolism, have been detected on the fungal cell surface acting as adhesins that facilitate the interaction with host extracellular matrix components [50]. Enzymes associated with cell wall remodeling are restricted to three chitin synthases in Table 2, although five were detected in Vev. Chitin synthase G1CCK1 drew our attention for being highly enriched (FCs above 4.0) in Vev, VevOxi, and VevNO, absent in Vpb18, and Nt-acetylated in Vev, but not in Aev. This enzyme was also enriched in extracellular vesicles from *Paracoccidioides* Pb18 isolated from mice granulomas [18]. Chitin synthases belong to a family of virulence factors involved in cell wall synthesis and remodeling, which are essential for fungal cell viability and infection [51]. In *P. brasiliensis*, seven classes of chitin synthases have been described [52] and some are differentially expressed during the mycelium-to-yeast transition, which is a critical stage for fungal pathogenicity [53]. We do not know which of these chitin synthases correspond to the ones presently detected, especially C1GGK1.

Proteins related to stress response and antioxidant defense were numerous among the virulence factors prevalent in Vev (Table 2). Over half of the heat shock proteins validated across the proteomes were Vev-enriched and tended to be Nt-acetylated in all the samples, e.g., Hsp60 and Hsp90, that were also enriched in VevOxi and VevNO (Table 2). Among the antioxidants, there are four thioredoxin-like proteins (FCs > 7.96), where Nt-Ac varied from present in Vev, but absent from either Aev (C1G7E0) or Vpb18 (C1G6F9). We found a total of nine thioredoxin-like proteins across the proteomes and six are present in vesicles. Out of three superoxide dismutases across proteomes, two are Vev-enriched/unique (C1G4T8, C1GJI2). Only one glutathione peroxidase (C1GC65) was detected across proteomes, which was Vev-nriched, HAP, and Nt-acetylated only in vesicles. Out of three catalases, C1G035 was not HAP and Nt-Ac occurred only intracellularly (Table 2); one was enriched in Aev, VevOxi/VevNO, and Nt-acetylated in all samples (C1G0D4), while catalase C1GCL8 was enriched in VevOxi/VevNO, but not detected in the Vev vs. Aev proteome. Together, these findings show that antioxidant and stress-response proteins are highly delivered through *Paracoccidioides* extracellular vesicles and tend to be enriched in Vev, where they may promote fungal evasion from the host’s protective attack.

Regulatory, signal transduction, cell communication proteins, and transcription factors could be involved with the phenotypic shift in Apb18 yeasts mediated by co-incubation with Vev [16]. Across the proteomes, we found a series of GTP-binding proteins, including Rab, Ran, and Ras. Most of them were detected in vesicles, especially in Vev, and many have been associated with virulence (Table 2). In Table 2, these proteins are under signal transduction/cellular communication and genetic information processing/regulation terms. GTP-binding proteins Rho3 (C1GFK5) and Ras-like protein Rab7, or Ypt7 (C1GEC2), are involved in regulating vesicle trafficking and endosomal transport [54], which are essential for cellular homeostasis and signaling.

The present proteomic analysis also revealed a broad diversity of proteins directly or indirectly associated with transcription in extracellular vesicles, which are not listed in Table 2. Across proteomes, we identified 105 sequences associated with chromatin modification, RNA processing and splicing, with only 58 observed in Vpb18, versus 23 Vev-enriched/unique out of 36 detected in Vev, while 19 proteins were validated exclusively in vesicles (Vev and/or Aev). This is a remarkable finding. Transcription factors like BZIP (C1G1W2) and BYE1 (C1GN69) are related to stress response and were uniquely found in Vev. The vesicle secretion of such proteins may play a crucial role in pathogenesis and phenotypic transfer to other fungal cells by modulating gene expression and RNA processing in the recipient cell. Together, the finding of numerous regulatory, signal transduction, cell communication, and especially transcription-associated proteins may explain the long-term phenotypic shift achieved when incubating fungal EVs with recipient fungal cells [16,55,56], in addition to the already reported participation of regulatory small RNA [57].

The group of Vev-enriched virulence factors involved in transport and secretion was surprising (Table 2). These proteins were also found enriched in VevOxi and VevNO, half were Nt-acetylated only in Vev, and they were rarely detected in the Vpb18 cell proteome, possibly because they are mostly transmembrane by nature. The ABC multidrug transporter SidT (also termed leptomycin B resistance protein pmd1, C1FZR3) is a 142-kDa transmembrane transporter detected at high amounts only in Vev, where it is Nt-acetylated and phosphorylated. This protein resembles mammalian multidrug resistance (mdr) proteins and is associated with antifungal resistance in *Schizosaccharomyces pombe*, promoting resistance not only to leptomycin B, but also to other cytotoxic agents [58,59]. In this context, ABC multidrug transporter enrichment in *Paracoccidioides* Vev suggests it may be actively released in the Vev surface to facilitate detoxification, possibly through sequestration or efflux of toxic compounds at extracellular sites [60]. This mechanism is likely enhanced under sublethal oxidative and nitrosative stress, considering the protein was enriched in VevOxi and VevNO, reinforcing the role of EV-mediated transporter delivery in adaptive fungal responses. Also note in Table 2 the presence of Vev-unique kynurenine formamidase (C1G4J8), known to be necessary for the elimination of toxic metabolites in *S. cerevisiae* [61].

## 4. Discussion

In our previous characterization of the *P. brasiliensis* Vpb18 secretome, we identified 205 proteins in the extracellular vesicle’s fraction, including a series of potential virulence factors associated with cell wall remodeling, signaling, transport, response to stress, besides moonlight proteins with primary functions related to carbohydrate and protein metabolism [7]. Later, Castilho et al. [20] reported seminal differences between the proteomes of the yeast phase from *P. brasiliensis* Vpb18 and its attenuated variant Apb18: molecules related to fungal virulence, including antioxidant components associated with fungal escape from the immune system, were predominant in Vpb18. We presently show that the proteomes of extracellular vesicles derived from these fungal variants, respectively, named Vev and Aev, are also distinct. Among 145 fungal virulence-associated proteins detected in the Vev proteome, 64% were Vev-enriched/unique relative to Aev, while 78% of the virulence factors detected in Vpb18 yeast extracts were also delivered in Vev. These data strongly support the notion that extracellular vesicles from virulent *P. brasiliensis* selectively concentrate proteins that may play essential roles in *Paracoccidioides* pathogenesis when safely delivered to the host’s environment. Our work presents original data in human pathogenic fungi concerning the modulation of vesicle protein cargo due to sublethal oxidative and nitrosative stress. In addition, our data significantly advances the field of extracellular vesicle proteomics by also exploring the *N*-terminal acetylation (Nt-Ac) of the identified proteins in the virulent/attenuated model to a dimension that has scarcely been investigated.

We found a higher proportion of Nt-acetylated proteins in Vev than in Vpb18 yeasts and an unexpected variation in Nt-acetylation of the same protein in distinct samples. Proteins like 5-oxiprolinase (C1GD55), fumarylacetoacetase (C1GMH9), mitochondrial succinate-CoA ligase (C1G0C7), catalase (C1G035), glutathione peroxidase (C1GC65), superoxide dismutase (C1G4T8), thioredoxin domain-containing protein (C1G6F9), aspartate aminotransferase (C1G388), glucose 6-phosphate isomerase (C1G0R1), malato synthase (C1GCI0), triosophosphate isomerase (C1G120), histone H2B, and elongation factor Tu (C1GGI0) were Nt-acetylated in either Vev or Vpb18 parent cells, suggesting that Nt-acetylation may have a role in protein compartmentalization. Other Vev-enriched virulence factors like chitin synthase (C1GGK1), 5-oxiprolinase (C1GD55), phosphoenolpyruvate carboxykinase (C1GML7), thioredoxin domain-containing protein (C1G7E0), aspartate aminotransferase (C1G3V5), carbonic anidrase (C1G0C5), and zinc/iron permease (C1GGI0) were Nt-acetylated in either Vev or Aev, suggesting that Nt-acetylation could protect these enzymes against degradation and/or fine-tune their activity to an optimal level. In humans, e.g., the Nt-acetylation of phosphoenolpyruvate carboxykinase prompts proteasomal degradation [62]. Although we found proteasome components in vesicles, we cannot assure they are active against target proteins. In counterpart, the possibility of Nt-acetylation occurring inside the EVs cannot be discarded, since we found an *N*-terminal acetyltransferase Nat1 (C1G4C0) in both Vev and Aev vesicle samples, while Nt-deacetylases were not detected in the present EV proteomes. However, these assumptions deserve further experimental validation.

In the present work, transmembrane proteins followed the general trend of being detected more abundantly in EVs than in the parent cells [41]. Although EV surface markers are extremely useful for EV detection and isolation, fungal EV markers are not easy to find and have rarely been suggested [41,63]. We can point out five transmembrane proteins that were detected at high amounts uniquely in Vev and Aev, but not in the parent cell extracts: potassium/sodium efflux P-type ATPase (C1G3T6), ZIP family zinc transporter (C1GEX7), GPR1 FUN34 yaaH family protein (C1GN52), MFS domain-containing protein (C1G1D9), and copper transport (C1GBT3). Of these, the GPR1 FUN34 yaaH family protein does not share significant homology with other proteins and could be considered for further investigation of a molecular signature for *Paracoccidioides* vesicles.

The cell wall polysaccharide structure has a fundamental role in cell survival and interaction with the innate immune system [64]. Fungal EVs carry enzymes involved in cell wall synthesis and remodeling, suggesting that they can mediate EV traversal through this compartment [7,65,66,67]. Knockout *S. cerevisiae* mutants for beta-1,3-glucan synthase or chitin synthase released increased amounts of EVs, possibly because the cell wall was defective and hence more porous [68]. In *C. neoformans*, mutants for eight chitin synthase genes presented unexpected effects in extracellular vesicle yields [69]. The chs3 mutant, that has the highest cell wall alterations, produced fewer EVs than the chs4 and chs5 mutants, which preserve the cell wall structure. In *H. capsulatum*, but not in *C. albicans*, caffeine, an inhibitor of chitinases, negatively affected the EV yields and chitinolytic activity [70]. Therefore, *H. capsulatum* EV chitinases seem to play an active role in facilitating the EV transport through the cell wall. Chitinase has not been detected in extracellular vesicles from *P. brasiliensis* Vpb18 in the present or previous works [7,18], but it was found soluble in the EV-free fraction of the secretome [7]. We presently observed that three chitin synthases, a chitin synthase export chaperone (C1G4V8), and an alpha-1,2-mannosidase (C1G9X5) were Vev-enriched . Other cell wall-related enzymes were more abundant in Aev from attenuated *P. brasiliensis*, specifically: 1,3-beta-glucanosyltransferase (C1GBS5), alpha-1,3-glucan synthase (C1G7L4), alpha-mannosidase (C1GA62), mannosidases (C1GCN9, C1GBX4), mannosyltransferases (C1GLD7, C1G435), and mannose-1-phosphateguanyltransferases (C1G065, C1GH87), besides a beta-1,3-glucanase (C1G6Q7) that was enriched in the stress VevOxi and VevNO samples. We could speculate that the more virulent the isolate, the more it holds cell wall-related enzymes in the cell to rapidly remodel the cell wall to evade the immune system and/or stress conditions.

Our group had the opportunity to compare three *Paracoccidioides* Vev proteomes carried out years apart, specifically, the most recent Vev (Figure 1), Vev control of the stress proteomes (Figure 7), and Vev fraction of the secretome [7]. For vesicle production, we systematically used a Vpb18 fungal isolate that has continuously been recovered from mice organs and we cultivated the yeasts in Ham/glc medium (either liquid or solid) for one (stress proteomes) or two days. Among the punctual differences we observed when comparing these three Vev proteomes, we highlight the absence of the secreted gp43 glycoprotein (beta-1,3-glucanase, PADG_07615) in the present work, although it has earlier been detected in both EV (at low amounts) and EV-free fractions of the Vpb18 secretome [7]. In counterpart, gp43 was enriched in the EV proteome recently analyzed by Montanari Borges and colleagues [18], who also detected a beta-1,3-glucan synthase that was not present in the EV proteomes analyzed by our group. The gp43 is a potent diagnostic and protective *P. brasiliensis* antigen, which has extensively been studied as a prophylactic and immunotherapeutic vaccine due to the proinflammatory properties concentrated in the internal P10 peptide [71]. Nevertheless, it has eventually been reported as a negative immune modulator [13] and a virulence factor due to its adhesive properties [72]. Therefore, the presence of gp43 in extracellular vesicles would definitely make a difference in the experimental data variation between research groups.

It is well accepted that the EV cargo can be modulated by environmental conditions [1], and the examples in pathogenic fungi are growing. *H. capsulatum* and *C. neoformans* vesicle cargo varied with the complexity of the culture media, in a way that EVs produced in nutritionally restrictive medium were richer in molecules associated with virulence [73,74]. The *H. capsulatum* EV content was also modulated by monoclonal antibodies anti-Hsp60, which bind to the fungal surface, when added to the culture medium in a dose-dependent manner [75]. Modulation of EV cargo also occurs in different forms of the same species, e.g., planktonic *C. albicans* EVs significantly differ from biofilm EVs [76], while the differences in EV biofilm cargo seem to be more prominent with the *Candida* phylogenetic distance of the parent cells [77]. We presently show original results of protein cargo modulation in extracellular vesicles produced by *P. brasiliensis* under sublethal oxidative and nitrosative stress. In mammalian systems, cells under oxidative stress release EVs containing signaling molecules capable of positively or negatively influencing the redox status of distant cells and tissues [78]. By using mild stress conditions in order to avoid cell damage and artifact in vesicle preparation, we believe that we fortuitously analyzed “primed” EVs from cells that were preemptively activating anticipatory responses to stress [42]. By releasing EVs with “pre-selected content”, one can envision that fungi could communicate environmental changes to recipient fungal cells, promoting the activation of conserved protective pathways even before encountering actual stresses, such as phagocyte attacks or oxidative stress.

Together, our findings highlight the relevance of our fungal model to unravel the significance and nuances of fungal extracellular vesicles in fungal pathogenesis and phenotypic transfer.

## 5. Conclusions

The overlap of stress-related proteins in EVs produced under nitrosative and oxidative stress conditions with those enriched/unique in Vev provides strong support to the notion that *Paracoccidioides brasiliensis* EVs are not random reflections of the cellular proteome, but rather structured entities with selective cargo composition and post-translational modifications that may have a role in vesicle localization and protein function. Our findings highlight the relevance of our fungal model to study the nuances of fungal EV protein cargo and their functionality.

## Figures and Tables

**Table 1:** Enriched proteins in VevOxi and/or VevNO that were also enriched in Vev relative to Aev. Virulence-associated proteins (VR) and abundance fold changes are shown.

Protein Accession No.	Protein Description	FC * VevOxi	FC * VevNO	FC Vev/Aev	VR
Carbohydrate metabolism					
C1G8R5	6-phosphogluconate dehydrogenase, decarboxylating	2.97	1.51	8.41	
C1G0C5	Carbonic anhydrase	2.43	0.87	4.62	Yes
C1G1C8	Fructose-bisphosphate aldolase	2.25	2.75	1.82	Yes
C1G0R1	Glucose-6-phosphate isomerase	2.51	1.14	7.29	Yes
C1G5F6	Glyceraldehyde-3-phosphate dehydrogenase	5.31	3.12	1.97	Yes
C1G8V5	Glycosidase	1.81	5.1	Unique	Yes
A0A0A0HX82	Phosphoglucomutase (alpha-D-glucose-1,6-bisphosphate-dependent)	2.41	1.9	8.9	
C1G4N0	Phosphoglycerate kinase	2.53	1.63	1.84	Yes
C1GJI4	Transaldolase	1.72	0.84	Unique	
C1GC82	Transketolase	3.81	2.85	3.08	
Tricarboxylic acid (TCA) and glyoxylate cycles					
C1GLZ6	Citrate synthase	5.74	2	Unique	
C1GLB8	Malate dehydrogenase	2.3	1.21	1.59	
C1GCI0	Malate synthase	1.44	1.5	1.58	Yes
Lipid and phospholipid metabolism					
C1G421	Acetyl-CoA C-acyltransferase	1.79	1.3	1.35	
C1G2P3	Enoyl-CoA hydratase	5.58	2.1	4.55	Yes
C1GHR9	Short-chain 2-methylacyl-CoA dehydrogenase	2.48	1.18	3.43	
C1GKG2	Very-long-chain 3-oxoacyl-CoA reductase	2.13	1.57	1.31	
Energy metabolism and biosynthesis					
C1GIX7	Acetyltransferase component of pyruvate dehydrogenase complex	2.77	2.01	1.49	
C1G0P4	AMP-dependent synthetase/ligase domain-containing protein	2.28	3.07	2.03	
C1G5X3	ATP synthase subunit 4	1.95	1.13	Unique	
C1GCK7	ATP synthase subunit d, mitochondrial	3.03	1.88	2.75	
C1GN84	FAD-binding FR-type domain-containing protein	1.5	1.67	3.14	
C1GKG3	NAD(P)H:quinone oxidoreductase, type IV	3.63	3.81	1.61	
C1GJT8	Nucleoside diphosphate kinase	1.47	1.67	1.86	Yes
C1GML7	Phosphoenolpyruvate carboxykinase (ATP)	1.67	1.59	1.88	Yes
Amino acid and nucleotide metabolism					
C1GLT7	5-methyltetrahydropteroyltriglutamate--homocysteine S-methyltransferase	4.92	5.43	1.91	
C1G025	Aromatic-L-amino-acid decarboxylase	3.76	6.77	Unique	
C1G388	Aspartate aminotransferase	4.63	4.35	1.7	Yes
C1G9D5	Urease	1.84	1.14	2.38	Yes
Transport and secretion					
C1GEX3	ABC metal ion transporter	2.22	1.52	2.19	
C1G2Y7	ABC transporter domain-containing protein	2.69	2.89	2.92	Yes
C1G8S5	Amino acid permease/ SLC12A domain-containing protein	1.53	1.64	4.56	Yes
C1GA14	Clathrin heavy chain	0.84	1.74	1.93	Yes
C1GBT3	Copper transport protein	2.43	2.39	4.54	Yes
C1GK98	Chromate ion transporter	1.62	0.92	2.96	
C1FZR3	Leptomycin B resistance protein pmd1	1.83	2.39	Unique	Yes
C1GL34	Major facilitator superfamily (MFS) profile domain-containing protein	1.7	2.08	4.98	Yes
C1GJY9	Major facilitator superfamily (MFS) profile domain-containing protein	3.86	4.59	4.65	Yes
C1G1D9	Major facilitator superfamily (MFS) profile domain-containing protein	2.13	2.22	2.12	Yes
C1G7G9	Major facilitator superfamily (MFS) profile domain-containing protein	5.06	5.7	1.91	Yes
C1GM00	Plasma membrane ATPase	1.35	1.75	1.86	Yes
C1GGI0	Zinc/iron transporter	1.31	1.96	1.96	Yes
C1G7B3	YeeE/YedE family integral membrane protein	4.35	3.97	2.03	
Protein synthesis, processing and degradation					
C1G5J0	40S ribosomal protein S15	1.87	1.62	1.33	
C1GGR5	40S ribosomal protein S20	3.34	4.29	1.69	
C1G9U4	60S acidic ribosomal protein P0	1.87	1.52	2.16	
C1GKL7	60S ribosomal protein L12	2.87	2.62	Unique	
C1GC66	60S ribosomal protein L22	5.5	2.62	4.67	
A0A0A0HVH2	60S ribosomal protein L31	1.13	1.75	1.42	
A0A0A0HV09	ATP-dependent RNA helicase eIF4A	1.64	1.73	1.36	
C1GIV4	Proteasome subunit alpha type-2	2.2	1.17	Unique	
C1G9A5	Protein disulfide-isomerase	1.34	1.5	5.38	
Genetic information processing and regulation					
C1GF71	Histone H2B	2.57	2.03	1.31	Yes
A0A0A0HU09	K Homology domain-containing protein	2.81	2.17	1.9	
C1GEW2	SsDNA binding protein	2.27	3.81	2.97	
Stress response and antioxidant defense					
C1GC65	Glutathione peroxidase	2.48	1.84	2.84	Yes
C1GLX8	Hsp60-like protein	3.32	2.16	2.54	Yes
C1GKC9	Hsp90-like protein	2.45	1.81	1.83	Yes
C1G4T8	Superoxide dismutase	5.17	2.3	Unique	Yes
C1G7E0	Thioredoxin domain-containing protein	2.17	1.06	7.96	Yes
Cell wall biogenesis and remodeling					
C1GGK1	Chitin synthase	4.11	4.23	4.35	Yes
C1GBY7	GPI-anchored cell wall protein	2.31	2.08	Unique	Yes
Signal transduction and cellular communication					
C1G1C6	CSC1/OSCA1-like 7TM region domain-containing protein	1.11	1.36	4.27	
C1GCT8	GTP-binding nuclear protein	1.55	1.36	1.45	
C1GLU6	GTP-binding protein rhoA	1.46	1.46	1.4	Yes
C1GE03	Ras-like protein	1.31	1.21	1.65	Yes
C1GMU5	t-SNARE coiled-coil homology domain-containing protein	1.44	1.51	1.39	
Uncharacterized/unknown function					
C1GKF2	FAR-17a/AIG1-like protein	7.89	8.06	Unique	
C1GMY9	DUF1774-domain-containing protein	1.48	0.91	Unique	
C1G9G6	Uncharacterized protein	1.09	2.23	Unique	

* FC: Fold change in reference to the control condition.

**Table 2:** Fungal virulence-associated proteins that are Vev-enriched relative to Aev. Higher-abundance proteins (HAP) in VevOxi and VevNO are indicated, along with those showing *N*Ω-terminal acetylation (Nt-Ac) in each proteome are indicated.

Protein Accession No.	Protein Description	FC * Vev/Aev	HAP **		Nt-Ac ***		
			VevOxi	VevNO	Vev	Aev	Vpb18
Carbohydrate metabolism							
C1G0C5	Carbonic anhydrase	4.62	HAP		Nt-Ac	-	x
C1G1C8	Fructosexbisphosphate aldolase	1.82	HAP	HAP	Nt-Ac	Nt-Ac	Nt-Ac
C1G0R1	Glucose-6-phosphate isomerase	7.29	HAP		-	-	Nt-Ac
C1G0I9	Glucosidase 2 subunit beta	1.48			-	-	-
C1GL11	Glycerol-3-phosphate dehydrogenase [-D(+)]	Unique	x	x	-	x	x
C1G5F6	Glyceraldehyde-3-phosphate dehydrogenase	1.97	HAP	HAP	Nt-Ac	Nt-Ac	Nt-Ac
C1G8V5	Glycosidase	Unique	HAP	HAP	-	x	-
C1G801	Glycosyltransferase family 62 protein	Unique			-	-	-
C1G4N0	Phosphoglycerate kinase	1.84	HAP	HAP	Nt-Ac	Nt-Ac	Nt-Ac
C1GCX3	Phosphoglycerate mutase (2 3-diphosphoglycerate-independent)	Unique	x	x	-	x	-
C1GI20	Triosephosphate isomerase	2.83			-	-	Nt-Ac
Tricarboxylic acid (TCA) and glyoxylate cycles							
C1GCI7	Isocitrate lyase	6.89	x	x	-	-	-
C1GCI0	Malate synthase	1.58	HAP	HAP	Nt-Ac	Nt-Ac	-
C1G294	Succinate--CoA ligase [ADP-forming] subunit alpha mitochondrial	Unique	x	x	-	x	-
C1G0C7	Succinate--CoA ligase [ADP-forming] subunit beta mitochondrial	2.87	x	x	-	-	Nt-Ac
Lipid and phospholipid metabolism							
C1G2P3	Enoyl-CoA hydratase	4.55	HAP	HAP	-	-	-
C1GFA5	Phospholipase D	6.14	x	x	-	-	x
C1GA36	Phospholipase D/nuclease	Unique	x	x	-	x	x
C1G7F7	Lysophospholipase	Unique	x	x	-	x	x
Energy metabolism and biosynthesis							
C1G5E8	Acid phosphatase	4.74			-	-	x
C1GMH9	Fumarylacetoacetase	Unique	x	x	-	x	Nt-Ac
C1GJT8	Nucleoside diphosphate kinase	1.86	HAP		Nt-Ac	Nt-Ac	Nt-Ac
C1GML7	Phosphoenolpyruvate carboxykinase (ATP)	1.88	HAP	HAP	Nt-Ac	-	Nt-Ac
C1GM00	Plasma membrane ATPase	1.86	HAP	HAP	Nt-Ac	Nt-Ac	Nt-Ac
Amino acid and nucleotide metabolism							
C1GD55	5-oxoprolinase	26.74	x	x	Nt-Ac	-	-
C1G388	Aspartate aminotransferase	1.7	HAP	HAP	-	-	Nt-Ac
C1G3V5	Aspartate aminotransferase	2.31	x	x	Nt-Ac	-	Nt-Ac
C1GJM4	Aspartyl aminopeptidase	Unique	x	x	-	x	-
C1G022	Hydantoinase	Unique	x	x	-	x	x
C1G4J8	Kynurenine formamidase	Unique	x	x	-	x	-
C1G312	Ornithine aminotransferase	Unique	x	x	-	x	-
C1G9D5	Urease	2.38	HAP		-	-	-
Transport and secretion							
C1FZR3	ABC multidrug transporter SidT	Unique	HAP	HAP	Nt-Ac	x	x
C1G2Y7	ABC transporter domain-containing protein	2.92	HAP	HAP	-	-	x
C1G8S5	Amino acid permease/SLC12A domain-containing protein	4.56	HAP	HAP	Nt-Ac	-	x
C1GA14	Clathrin heavy chain	1.93		HAP	Nt-Ac	-	-
C1GBT3	Copper transport protein	4.54	HAP	HAP	-	-	x
C1GL34	Major facilitator superfamily (MFS) profile domain-containing protein	4.98	HAP	HAP	Nt-Ac	-	-
C1GJY9	Major facilitator superfamily (MFS) profile domain-containing protein	4.65	HAP	HAP	Nt-Ac	-	x
C1G1D9	Major facilitator superfamily (MFS) profile domain-containing protein	2.12	HAP	HAP	-	-	x
C1G7G9	Major facilitator superfamily (MFS) profile domain-containing protein	1.91	HAP	HAP	-	-	x
C1GIS2	Zinc/iron permease	Unique	x	x	-	x	x
C1GGI0	Zinc/iron permease	1.96	HAP	HAP	Nt-Ac	-	-
Protein synthesis, processing and degradation							
C1G4T3	Elongation factor Tu	Unique	x	x	-	x	Nt-Ac
C1GJI6	Peptidase S8/S53 domainxcontaining protein	1.93	x	x	-	-	x
C1G3B6	Peptidyl-prolyl cis-trans isomerase	Unique	x	x	-	x	x
C1GA06	Peptidyl-prolyl cis-trans isomerase	15.4	x	x	-	-	-
C1GD67	Peptidyl-prolyl cis-trans isomerase	Unique	x	x	-	x	-
C1GKU6	Peptidyl-prolyl cis-trans isomerase	1.45	x	x	-	-	-
C1GGQ1	Peptidylprolyl isomerase	1.68			-	Nt-Ac	Nt-Ac
C1FYT6	Zinc metalloproteinase	14.88	x	x	-	-	x
Genetic information processing and regulation							
C1G092	GTP-binding protein ypt2	3.93	x	x	-	-	-
C1GF71	Histone H2B	1.31	HAP	HAP	Nt-Ac	Nt-Ac	-
C1FYR1	Rab family other	2.66	x	x	Nt-Ac	Nt-Ac	Nt-Ac
C1FZK1	Ran GTPase-activating protein 1	1.52	x	x	-	-	-
Stress response and antioxidant defense							
C1G445	Aha1_N domain-containing protein	1.57	x	x	-	-	-
C1G035	Catalase	3.53			-	-	Nt-Ac
C1G2G2	Glutaredoxin domain-containing protein	Unique	x	x	-	x	x
C1GC65	Glutathione peroxidase	2.84	HAP	HAP	Nt-Ac	Nt-Ac	-
C1GAU3	Heat shock protein STI1	2.77	x	x	-	-	-
C1GLX8	Hsp60-like protein	2.54	HAP	HAP	Nt-Ac	Nt-Ac	Nt-Ac
C1G0P0	Hsp7-like protein	1.74			-	-	Nt-Ac
C1GLI2	Hsp72-like protein	2.13			Nt-Ac	Nt-Ac	Nt-Ac
C1G6F6	Hsp75-like protein	2.6	x	x	Nt-Ac	Nt-Ac	Nt-Ac
C1GKC9	Hsp90-like protein	1.83	HAP	HAP	Nt-Ac	Nt-Ac	Nt-Ac
C1G1M5	Hsp98-like protein	1.44	x	x	-	-	-
C1G532	Nitroreductase domainxcontaining protein	3.2	x	x	-	-	x
C1GBB1	Redoxin domain-containing protein	Unique	x	x	-	x	-
C1G9M7	SHSP domain-containing protein	Unique	x	x	-	x	-
C1G4T8	Superoxide dismutase	Unique	HAP	HAP	-	x	Nt-Ac
C1GJI2	Superoxide dismutase [Cu-Zn]	1.7	x	x	-	-	-
C1GE86	Survival factor 1	1.46	x	x	-	-	-
C1GKT9	Thioredoxin domain-containing protein	Unique	x	x	-	x	-
C1G6F9	Thioredoxin-like fold domain-containing protein	12.26			Nt-Ac	Nt-Ac	-
C1GDK8	Thioredoxin domain-containing protein	8.44	x	x	-	-	-
C1G7E0	Thioredoxin domain-containing protein	7.96	HAP		Nt-Ac	-	Nt-Ac
Cell wall biogenesis and remodeling							
C1GGK1	Chitin synthase	4.35	HAP	HAP	Nt-Ac	-	x
C1GKQ4	Chitin synthase	1.9			-	-	-
A0A0A0HU35	Chitin synthase C	Unique	x	x	-	x	x
C1GBY7	GPI-anchored cell wall protein	Unique	HAP	HAP	-	x	x
Cellular organization							
C1FZC6	Actin cytoskeleton-regulatory complex protein PAN1	4.21	x	x	-	-	x
C1GII4	Septin-type G domain-containing protein	1.63			Nt-Ac	Nt-Ac	Nt-Ac
Signal transduction and cellular communication							
C1GBU2	CK1/CK1/CK1-G protein kinase	2.02	x	x	Nt-Ac	Nt-Ac	x
C1GFK5	GTPxbinding protein rho3	Unique	x	x	-	x	x
C1GLU6	GTPxbinding protein rhoA	1.4	HAP	HAP	Nt-Ac	Nt-Ac	Nt-Ac
C1GJ63	High osmolarity signaling protein SHO1	Unique	x	x	-	x	x
C1GET1	Non-specific serine/threonine protein kinase	5.6	x	x	-	-	-
C1G6S5	Non-specific serine/threonine protein kinase	3.66	x	x	-	-	-
C1FYN4	Non-specific serine/threonine protein kinase	2.38	x	x	Nt-Ac	-	-
C1GBG9	Non-specific serine/threonine protein kinase	2.19	x	x	-	-	-
C1GM91	Ras-GAP domainxcontaining protein	3.44	x	x	-	-	-
C1GE03	Ras-like protein	1.65	HAP		-	-	-
C1GEC2	Ras-like protein Rab7	Unique	x	x	-	x	-

* FC: Fold change. ** “x” indicates that the protein was not detcted or validated. *** “-” indicates that Nt-Ac modification was not detected.

## Data Availability

The data presented in this study are openly available in ProteomeXchange Consortium [https://www.proteomexchange.org/] [PXD065468].

## Supplementary Materials

The following supporting information can be downloaded at: https://www.mdpi.com/article/10.3390/jof11100751/s1, Figure S1: Enrichment analysis of validated Vev and Aev proteins relative to their frequency in the Paracoccidioides brasiliensis Pb18 genome (UP000001628); Figure S2: Morphological and physicochemical properties of VevOxi and VevNO proteomes produced under sublethal oxidative and nitrosative stress; Figure S3. Enrichment analysis of validated VevOxi and VevNO proteins relative to their frequency in the Paracoccidioides brasiliensis Pb18 genome (UP000001628). Table S1. Enriched proteins in VevOxi and/or VevNO that were not enriched in Vev relative to Aev. Virulence regulators (VirReg) and abundance fold changes are shown.

## Abbreviations

Aev	Extracellular vesicles isolated from the Pb18 attenuated variant
Apb18	Paracoccidioides brasiliensis Pb18 attenuated variant
CFU	Colony forming units
DLS	Dynamic Light Scattering
EVs	Extracellular vesicles
FDR	false discovery rate
GO	Gene Ontology
HAP	High-abundance proteins
KEGG	Kyoto Encyclopedia of Genes and Genomes
Nt-Ac	Protein Nt-acetylation
NTA	Nanoparticle Tracking Analysis
PBS	Phosphate-buffered saline
PCM	Paracoccidioidomycosis
PdI	olydispersity index
PdI	polydispersity index
Pho	Phosphorylation
PTMs	Post-translational modifications
SEM	Scanning electron microscopy
SERS	Surface-enhanced Raman scattering
TEM	Transmission Electron Microscopy
Vev	Extracellular vesicles isolated from the Pb18 virulent variant
VevNO	Vev derived from yeast cells cultivated under sublethal nitrosative stress
VevOxi	Vev derived from yeast cells cultivated under sublethal oxidative stress
Vpb18	Paracoccidioides brasiliensis Pb18 virulent variant
ZP	zeta potential
