# Supplementary material for: Virulence and Stress-Related Proteins Are Differentially Enriched and N-Terminally Acetylated in Extracellular Vesicles from Virulent Paracoccidioides brasiliensis
Source: J Fungi (Basel). 2025 Oct 21;11(10):751. doi: 10.3390/jof11100751 (PMC12565617; doi:10.3390/jof11100751)

Table S1. Enriched proteins in VevOxi and/or VevNO that were not enriched in Vev relative to Aev. Virulence regulators (VirReg) and abundance fold changes are shown.

Protein accession no.	Protein description	FC* VevOxi	FC* VevNO	VirReg
**Carbohydrate metabolism**				
C1G440	Fructose-bisphosphatase	2.08	1.39	
C1G7G3	Glucose-6-phosphate 1-epimerase	4.56	5.98	
C1G2W2	Pyruvate kinase	3.63	1.61	
**Tricarboxylic acid (TCA) and glyoxylate cycles**				
C1GAG3	Isocitrate dehydrogenase [NADP]	12.64	8.69	
**Lipid and phospholipid metabolism**				
C1GCH9	Enoyl reductase (ER) domain-containing protein	1.43	2.97	
C1G065	Malonyl-CoA:ACP transacylase (MAT) domain-containing protein	8.75	14.32	
C1GMZ1	Peroxisomal hydratase-dehydrogenase-epimerase	13.36	9.92	
C1GJS2	Phosphatidylinositol transfer protein SFH5	1.11	1.39	
**Energy metabolism and biosynthesis**				
C1GIF6	ATP synthase subunit 5, mitochondrial	1.86	1.26	
C1GGJ2	Ketol-acid reductoisomerase, mitochondrial	1.7	0.91	
C1G3P8	Methylmalonate-semialdehyde dehydrogenase (CoA acylating)	1.93	2.55	
**Amino acid and nucleotide metabolism**				
C1GHD5	3-isopropylmalate dehydrogenase	2	1.57	
C1G4M0	Adenosylhomocysteinase	3.46	3.97	
C1GHS5	Amidase domain-containing protein	1.63	1.51	
C1GAT1	Amidase domain-containing protein	1.72	1.06	
C1FYN6	Amine oxidase domain-containing protein	6.41	1.6	
C1G7J4	Aminopeptidase	2.27	1.05	Yes
C1GLY5	Aspartate-semialdehyde dehydrogenase	6.77	2.91	
C1GA81	Aspartyl aminopeptidase	1.37	0.82	Yes
C1GBZ4	Glutamate dehydrogenase	1.44	2.08	
C1FYE6	Glutaminase	1.75	1.61	
C1G020	Glycine cleavage system P protein	2.33	0.99	
C1G0F9	Kynureninase	4.06	1.28	
C1GCX5	Serine hydroxymethyltransferase	2.28	1	Yes
C1GDE1	Serine hydroxymethyltransferase	1.36	0.88	Yes
**Transport and secretion**				
C1G3C4	ADP/ATP translocase	2.83	1.99	Yes
C1GMQ0	Amino acid permease/ SLC12A domain-containing protein	1.62	1.64	Yes
C1GAF5	Coatomer subunit alpha	1.85	4.35	
C1GLM2	Mitochondrial outer membrane protein porin	2.91	1.97	Yes
C1GHD6	P-type Na(+) transporter	1.96	1.67	
C1G3T6	P-type Na(+) transporter	1.14	1.32	
C1FYW7	Peptide transporter PTR2	1.03	1.4	
C1GGB6	Transmembrane 9 superfamily member	2.22	1.67	
**Protein synthesis, processing and degradation**				
C1GG76	40S ribosomal protein S18	1.33	1.31	
C1GMV8	40S ribosomal protein S2	2.55	2	
C1GHE4	40S ribosomal protein S22	1.61	1.72	
C1G391	40S ribosomal protein S3	1.66	1.4	
C1G810	40S ribosomal protein S4	1.72	1.52	
C1GHV2	40S ribosomal protein S5	1.91	1.94	
C1G3Y8	40S ribosomal protein S6	3.73	3.2	
C1FYR6	40S ribosomal protein S7	4.41	5.21	
C1G820	60S ribosomal protein L21-A	1.87	1.82	
A0A0A0HYV6	60S ribosomal protein L5	2.04	1.47	
C1GGC2	Cysteine proteinase 1, mitochondrial	1.54	1.57	

C1GLI9	Elongation factor 2	1.49	1.3	
C1GEN5	Large ribosomal subunit protein uL4 C-terminal domain-containing protein	1.56	1.55	
A0A0A0HWY2	Large ribosomal subunit protein uL6 alpha-beta domain-containing protein	3.32	2.46	
C1GG53	Proteasome alpha-type subunits domain-containing protein	1.62	1	
C1G9P6	Proteasome alpha-type subunits domain-containing protein	1.92	1.24	
C1G7N7	Proteasome component PUP2	1.34	2.14	Yes
C1G175	Proteasome subunit alpha type	1.52	1.34	
C1GMF6	Proteasome subunit alpha type	1.76	0.9	
C1G8U4	Proteasome subunit beta	4.29	2.14	
C1G9M9	Proteasome subunit beta	2.6	1.02	
C1GLF1	Proteasome subunit beta	1.54	0.83	
C1G9N1	Proteasome subunit beta type-6	3.78	1.3	
C1G6M3	Ribosomal protein	1.52	1.49	
C1GGT8	Small ribosomal subunit protein eS1	1.59	1.36	
**Genetic information processing and regulation**				
C1G9F5	FCP1 homology domain-containing protein	2.03	3.14	
A0A0A0HUI8	Histone H2A.Z	1.41	1.29	Yes
C1GIP8	Histone H4	3.18	2.19	
C1G712	PH domain-containing protein	2.77	1.33	
A0A0A0HUX3	Proliferating cell nuclear antigen	2.75	0.95	
C1G647	RRM domain-containing protein	2.13	1.9	Yes
**Stress response and antioxidant defense**				
C1GCL8	Catalase	12.64	2.14	Yes
C1G0D4	Catalase	2.79	3.12	Yes
C1G8H6	Endoplasmic reticulum chaperone BiP	1.7	1.67	Yes
C1G7K8	Peroxidase	1.75	1.57	Yes
C1G349	Thioredoxin domain-containing protein	4.35	2.73	Yes
**Cell wall biogenesis and remodeling**				
C1G7L4	Alpha-1,3-glucan synthase	2.33	3.71	Yes
C1G6Q7	Glucan 1,3-beta-glucosidase	2.66	2	Yes
**Signal transduction and cellular communication**				
C1GB04	14-3-3 domain-containing protein	2.97	1.25	Yes
C1G9X0	14-3-3 family protein epsilon	2.17	1.18	Yes
C1GIN4	Annexin	14.12	25.11	
C1GKE7	Arp2/3 complex 34 kDa subunit	1.31	2.16	
C1FYX6	Band 7 domain-containing protein	1.42	1.72	
C1GBN1	CS domain-containing protein	1.59	0.78	Yes
C1G1Y1	Guanine nucleotide-binding protein alpha-3 subunit	5.13	6.32	
C1GLV1	GTP-binding protein ypt1 (Fragment)	1.89	1.84	Yes
C1GM08	GTP-binding protein ypt3	1.68	1.64	Yes
C1G2I2	Pheromone-processing carboxypeptidase KEX1	2.48	6.36	Yes
C1GC92	Prohibitin	5.43	3.01	
C1G2S7	Rab GDP dissociation inhibitor	7.01	14.72	Yes
C1G2J3	Septin-type G domain-containing protein	1.59	1.53	Yes
C1GCG8	V-SNARE coiled-coil homology domain-containing protein	2.46	2.97	
**Uncharacterized / unknown function**				
C1G1R3	Uncharacterized protein	1.73	1.58	
C1G8T9	Uncharacterized protein	1.55	4.29	
C1GDR6	Uncharacterized protein	2.36	1.91	
C1GHF6	Uncharacterized protein	2.03	1.57	
*FC: Fold change in reference to the control condition.				

Figure S1. Enrichment analysis of validated Vev and Aev proteins relative to their frequency in the *Paracoccidioides brasiliensis* Pb18 genome (UP000001628). We show selected terms according to Gene Ontology (GO) biological processes, KEGG and Reactome pathways, using STRING-db with an FDR cutoff of 0.05. Fold enrichment in each category represents the "% query" (proportion of detected proteins) divided by the "% universe" (proportion of proteins in the genome). The color scale represents $-\log_{10}$(FDR). The terms selected for graphical representations were chosen based on biological relevance, functional diversity, and broad coverage.

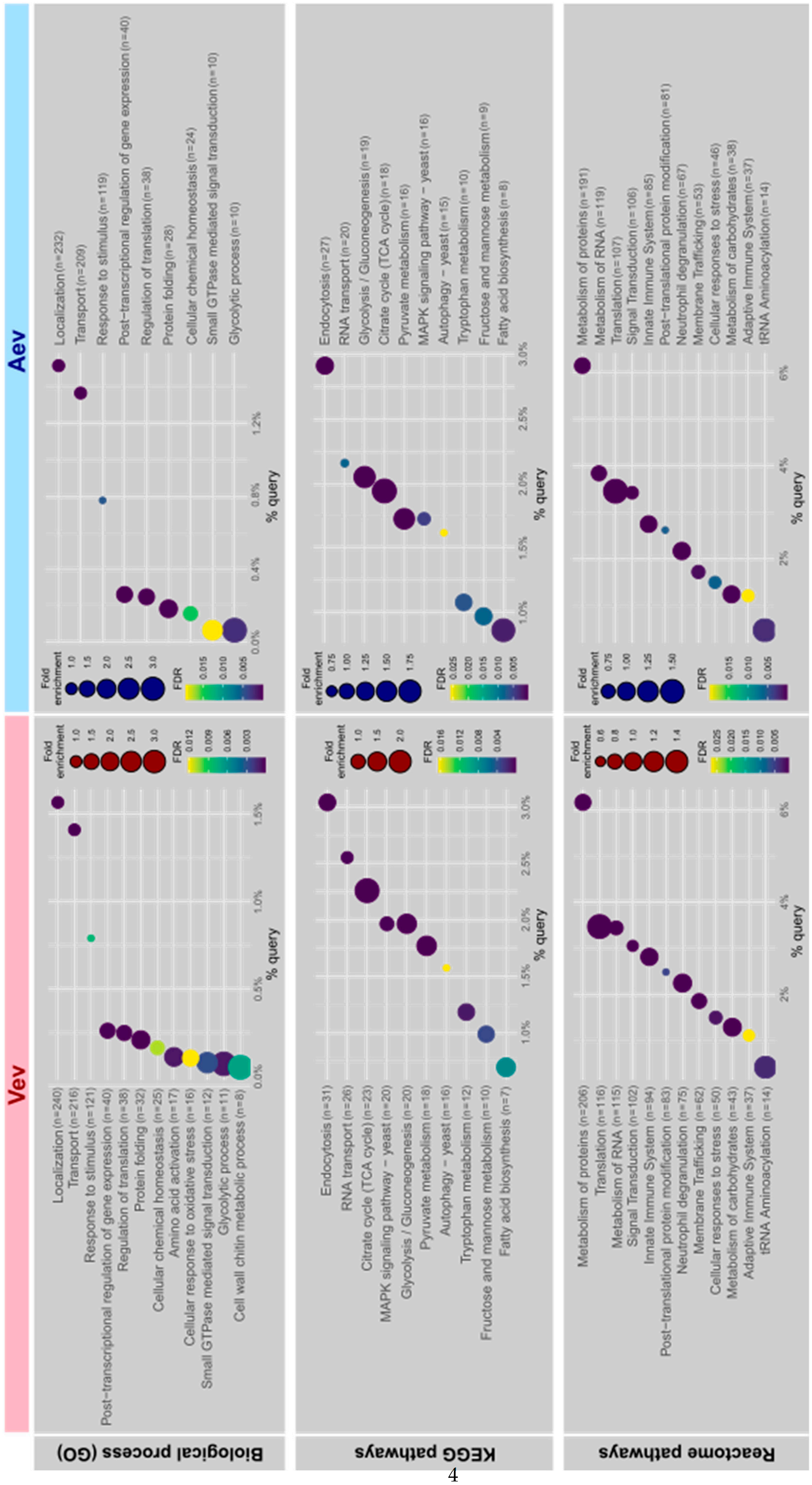

Vev
Aev
Biological process (GO)
KEGG pathways
Reactome pathways
Localization (n=240)
Transport (n=216)
Response to stimulus (n=121)
Post−transcriptional regulation of gene expression (n=40)
Regulation of translation (n=38)
Protein folding (n=32)
Cellular chemical homeostasis (n=25)
Amino acid activation (n=17)
Cellular response to oxidative stress (n=16)
Small GTPase mediated signal transduction (n=12)
Glycolytic process (n=11)
Cell wall chitin metabolic process (n=8)
% query
Fold enrichment
FDR
Endocytosis (n=31)
RNA transport (n=26)
Citrate cycle (TCA cycle) (n=23)
MAPK signaling pathway − yeast (n=20)
Glycolysis / Gluconeogenesis (n=20)
Pyruvate metabolism (n=18)
Autophagy − yeast (n=16)
Tryptophan metabolism (n=12)
Fructose and mannose metabolism (n=10)
Fatty acid biosynthesis (n=7)
Metabolism of proteins (n=206)
Translation (n=116)
Metabolism of RNA (n=115)
Signal Transduction (n=102)
Innate Immune System (n=94)
Post−translational protein modification (n=83)
Neutrophil degranulation (n=75)
Membrane Trafficking (n=62)
Cellular responses to stress (n=50)
Metabolism of carbohydrates (n=43)
Adaptive Immune System (n=37)
tRNA Aminoacylation (n=14)
Localization (n=232)
Transport (n=209)
Response to stimulus (n=119)
Post−transcriptional regulation of gene expression (n=40)
Regulation of translation (n=38)
Protein folding (n=28)
Cellular chemical homeostasis (n=24)
Small GTPase mediated signal transduction (n=10)
Glycolytic process (n=10)
Endocytosis (n=27)
RNA transport (n=20)
Glycolysis / Gluconeogenesis (n=19)
Citrate cycle (TCA cycle) (n=18)
Pyruvate metabolism (n=16)
MAPK signaling pathway − yeast (n=16)
Autophagy − yeast (n=15)
Tryptophan metabolism (n=10)
Fructose and mannose metabolism (n=9)
Fatty acid biosynthesis (n=8)
Metabolism of proteins (n=191)
Metabolism of RNA (n=119)
Translation (n=107)
Signal Transduction (n=106)
Innate Immune System (n=85)
Post−translational protein modification (n=81)
Neutrophil degranulation (n=67)
Membrane Trafficking (n=53)
Cellular responses to stress (n=46)
Metabolism of carbohydrates (n=38)
Adaptive Immune System (n=37)
tRNA Aminoacylation (n=14)

Figure S2. Morphological and physicochemical properties of VevOxi and VevNO proteomes produced under sublethal oxidative and nitrosative stress. (a) VevOxi and VevNO negative staining TEM images. A 200 nm-size bar is indicated. The insets show an amplified EV particle in each sample. b) Physicochemical parameters of VevOxi, VevNO, and Vev (control; Vev control for VevNO was produced at pH 5.5): NTA size distribution calculated from 10 independent preparations, where the D10, D50, and D90 parameters indicate the diameters below which 10%, 50%, and 90% of the EV population fit, respectively; hydrodynamic diameters (DLS) in nm, polydispersity index (PI), zeta potential (ZP) values expressed as the median of three measurements per replicate. *, statistically significant for $p < 0.05$; sterol and proteins contents.

(a)

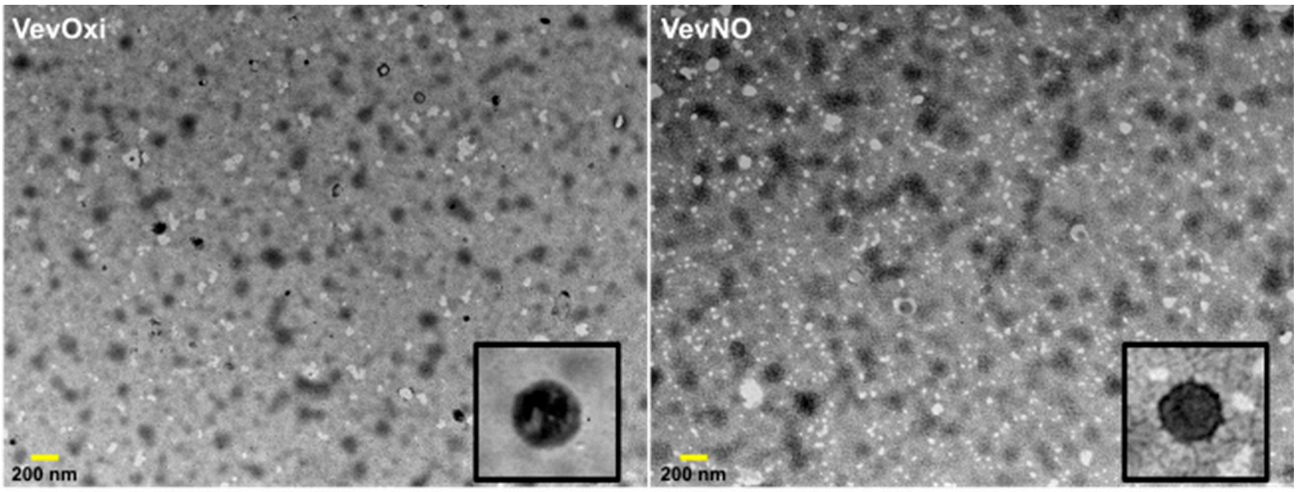

(b)

Method	Sample	Mean (nm)	Mode (nm)	SD (nm)	D10 (nm)	D50 (nm)	D90 (nm)
Nanoparticle tracking analysis (NTA)	**Vev**	62.3 ± 1.7	59.0 ± 0.9	17.3 ± 4.2	49.4 ± 0.8	59.1 ± 1.3	76.6 ± 5.6
	**VevOxi**	61.5 ± 0.2	59.1 ± 1.2	11.7 ± 1.1	51.9 ± 0.6	59.9 ± 0.7	71.0 ± 0.6
	**Vev**	62.5 ± 0.8	61.1 ± 2.4	14.1 ± 3.5	50.6 ± 0.7	61.1 ± 0.8	75.6 ± 5.13
	**VevNO**	79.3 ± 2.5	73.6 ± 1.4	22.6 ± 4.8	65.8 ± 0.4	74.1 ± 0.9	89.6 ± 5.0
Dinamic lightscattering (DLS)	**Sample**	**Mean (nm)**	**Mode (nm)**	**Polydispersity index (PdI)**	**Zeta potential (mV)**		
	**Vev**	160.1 ±18.5	132.7 ± 22.6	0.37± 0.05	-20.3 ± 3.4		
	**VevOxi**	213.2 ± 24.5*	153.4 ± 24.7	0.45 ± 0.07	-18.8 ± 3.8		
	**Vev**	181.1 ± 15.6	142.3 ± 23.5	0.37 ± 0.02	-22.1 ± 2.9		
	**VevNO**	172.9 ± 15.8	144.5 ± 20.2	0.36 ± 0.06	-19.2 ± 3.0		
Cholesterol and BCA protein assay	**Sample**	**Sterol (µM)**	**Protein (µg)**				
	**Vev**	22.97 ± 5.95	49.25 ± 25.85				
	**VevOxi**	12.28 ± 2.19*	32.13 ± 6.43				
	**Vev**	11.76 ± 3.69	28.25 ± 3.85				
	**VevNO**	8.76 ± 0.05	31.43 ± 14.93				

*Statistical significance was defined as $p < 0.05$ with a 95% confidence interval.

Figure S3. Enrichment analysis of validated VevOxi and VevNO proteins relative to their frequency in the *Paracoccidioides brasiliensis* Pb18 genome (UP000001628). We show selected terms according to Gene Ontology (GO) biological processes, KEGG and Reactome pathways, using STRING-db with an FDR cutoff of 0.05. Fold enrichment in each category represents the "% query" (proportion of detected proteins) divided by the "% universe" (proportion of proteins in the genome). The color scale represents $-\log_{10}$(FDR). The terms selected for graphical representations were chosen based on biological relevance, functional diversity, and broad coverage.

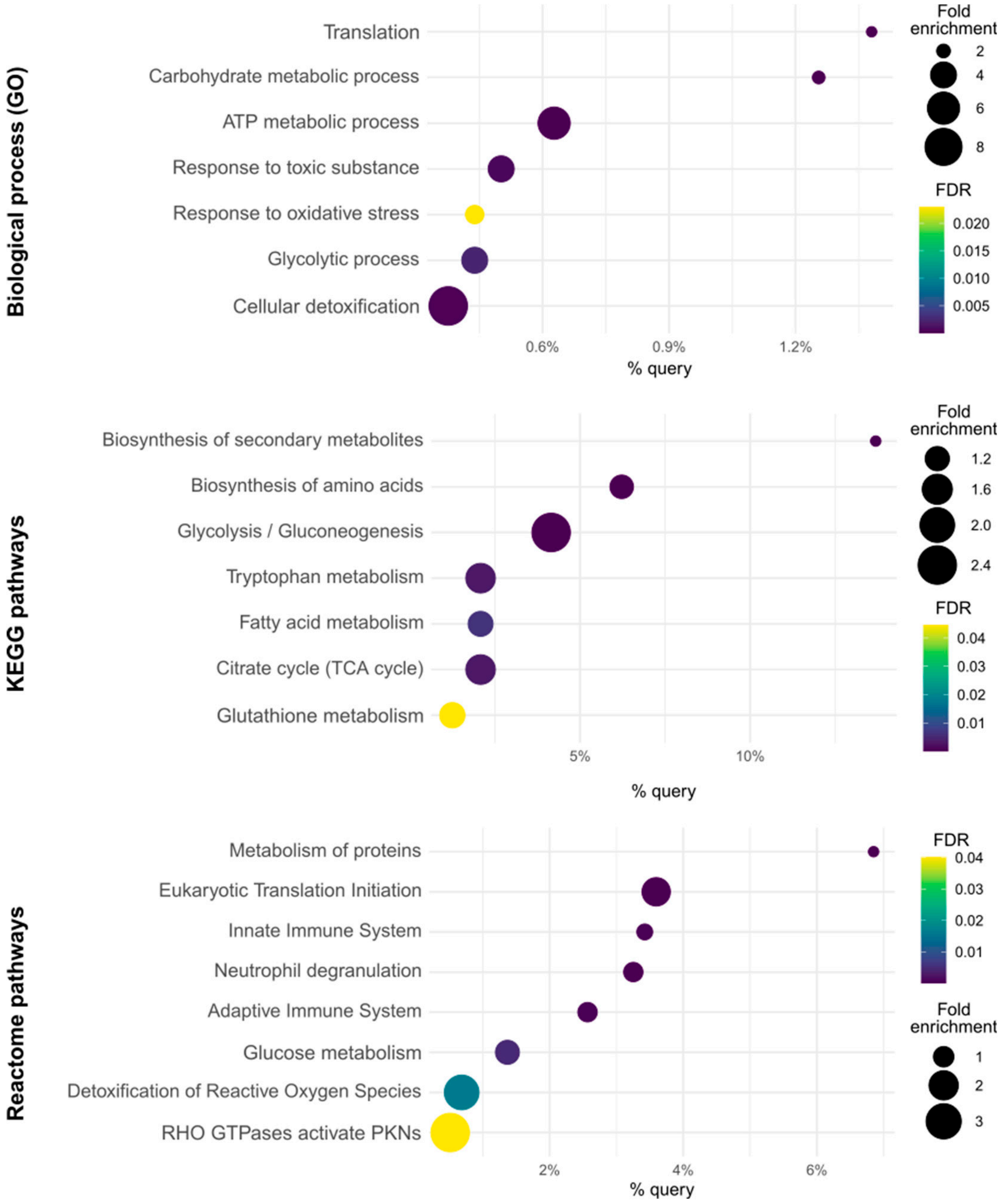

Supplement: Supplementary file 1 [file jof-11-00751-s001.zip › jof-3872265-supplementary.pdf]
